# Evolution of Avian Eye Size Is Associated with Habitat Openness, Food Type and Brain Size

**DOI:** 10.3390/ani13101675

**Published:** 2023-05-18

**Authors:** Yating Liu, Ying Jiang, Jiliang Xu, Wenbo Liao

**Affiliations:** 1 School of Ecology and Nature Conservation, Beijing Forestry University, Beijing 100083, China; 2 Key Laboratory of Southwest China Wildlife Resources Conservation (Ministry of Education), China West Normal University, Nanchong 637009, China; 3 Key Laboratory of Artificial Propagation and Utilization in Anurans of Nanchong City, China West Normal University, Nanchong 637009, China

**Keywords:** birds, eye size, brain size, ecology, evolution

## Abstract

**Simple Summary:**

Birds often exhibit differences in locomotion, foraging, and predator detection, many of which are often reflected in their eye sizes. Therefore, understanding the coevolutionary relationships between eye size and ecological factors, behaviours and brain size in birds is essential. Our results indicate that species with larger eye sizes reside in dense habitats, feed on invertebrates or vertebrates and have larger brains, suggesting that habitat openness, food type and cognition play critical roles in shaping visual sensitivity and resolution. However, we did not find any correlation between eye size and migration behaviour or foraging habitat, indicating that these factors are likely not major drivers of eye size evolution.

**Abstract:**

The eye is the primary sensory organ that obtains information from the ecological environments and specifically bridges the brain with the extra environment. However, the coevolutionary relationships between eye size and ecological factors, behaviours and brain size in birds remain poorly understood. Here, we investigate whether eye size evolution is associated with ecological factors (e.g., habitat openness, food type and foraging habitat), behaviours (e.g., migration and activity pattern) and brain size among 1274 avian species using phylogenetically controlled comparative analyses. Our results indicate that avian eye size is significantly associated with habitat openness, food type and brain size. Species living in dense habitats and consuming animals exhibit larger eye sizes compared to species living in open habitats and consuming plants, respectively. Large-brained birds tend to possess larger eyes. However, migration, foraging habitat and activity pattern were not found to be significantly associated with eye size in birds, except for nocturnal birds having longer axial lengths than diurnal ones. Collectively, our results suggest that avian eye size is primarily influenced by light availability, food need and cognitive ability.

## 1. Introduction

**Figure 1:**
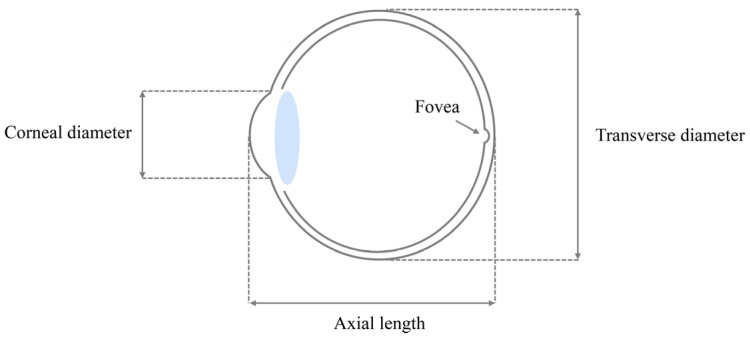
Schematic depicting the parameters taken for this study (transverse diameter and axial length) of the avian eye.

The eye is a vital sensory organ in animals, allowing them to obtain immediate and detailed information from their surrounding environment [1]. Light enters the eye and passes through several structures, including the cornea, anterior chamber, lens and vitreous humour before being captured by photoreceptors (rod cells and cone cells) in the retina [2,3,4,5]. Eyes with larger dimensions have wider pupil apertures, which allows for more light to enter [6]. This increased light-gathering ability can enhance visual sensitivity or/and acuity in low-light conditions, making larger eyes advantageous for certain visual tasks such as nocturnal or deep-sea vision [1,6]. However, the eye often trades-off between visual sensitivity and visual acuity, two fundamental capacities that are inherently conflicting [1,6,7]. Visual sensitivity depends on the pupil aperture and the size of the photoreceptors, and it is inversely proportional to the F-number [6,8]. The F-number is given by the pupil diameter divided by the focal length, and pupil diameter can be estimated by the corneal diameter and the focal length is approximately 0.6 times the axial length [6] (Figure 1). In contrast, visual acuity is determined by the focal length of the eye and the diameter of the photoreceptor [6]. Consequently, species need to trade-off between visual sensitivity and visual acuity to meet the practical needs of vision unless the volume of the eye is increased.

The eye’s ability to extract visual information from the environment depends on its size and dimensions [9]. A larger eye can accommodate a larger retina, allowing for more photoreceptors due to their organized and densely packed arrangement within the retina [1,10,11]. Thus, larger eyes with larger retinas and more photoreceptors have a greater capacity to capture visual information from the environment [1,2,9,12]. Obviously, an increase in eye volume confers either greater visual sensitivity or/and visual acuity [1,6,13]. Furthermore, a longer axial length is thought to be useful to animals that rely on vision for foraging or predator detection because a longer axial length increases the size of the image on the retina and allows for a greater number of photoreceptors to capture more detailed information [14,15,16].

Changes in environmental and ecological factors can impose pressures on variations in phenotypic characters and organ size in animals [17,18,19,20,21]. That optical environments predict the evolution of the eye has been the subject of significant attention in the literature [22,23,24,25]. In low photon environments, fishes, frogs and mammals have to increase the visual sensitivity of their eyes to maximize their chances of capturing photons, as evidenced by previous studies [7,26,27]. Consequently, a larger eye is better suited to capturing faint light compared to a smaller eye, as it can improve sensitivity [7,27]. Birds, like other vertebrates, can also adapt to low ambient light levels by developing larger eyes. For instance, a study conducted in an Amazonian rainforest found that large-eyed birds preferred to dwell in the deeper forest instead of on forest edges [28]. In addition, diurnal bird species dwelling in the darker understory and forested habitats possess larger eyes than species occupying nonforest habitats [25]. Birds with larger eyes also tend to begin their daily activities, such as singing or foraging, earlier in the morning than species with smaller eyes, indicating superior visual capabilities in low-light environments [29,30,31]. Similarly, nocturnal bird species have evolved adaptative traits (e.g., longer corneal diameters and axial lengths, and a higher degree of pooling of rod cells) to improve their visual abilities compared to their diurnal counterparts [15,32].

Birds’ large eyes indicate the significance of vision in guiding their behaviour [32,33,34]. For species foraging in a specific environment, the retina has undergone extraordinary adaptation in the number and distribution of photoreceptors to produce optimal resolution [35,36,37,38,39]. Previous studies have shown that moving prey (e.g., insects, voles) is more likely to be captured by birds with larger eyes [40]. The enlargement of eyes in animals can confer several benefits; however, this evolutionary adaptation comes with a cost. Enlarging eye size requires an increased metabolic investment to process neurological information, which can negatively affect other functional traits due to limited metabolic resources [41,42,43,44]. For instance, Hall and Heesy [16] found that there is a negative relationship between the relative eye volumes and flight speed of birds, indicating that large eyes can hinder their ability to fly at high speeds. Additionally, large eyes can also be more vulnerable to damage from disability glare or overexposure, which can impact foraging and predator detection [38,45,46]. Therefore, the evolution of eye size is a trade-off between the costs and benefits, and this trade-off’s extent is likely to vary between species depending on their ecological niche and selective pressures [7,25].

The development and evolution of sensory organs have been subjects of interest in the field of biology, particularly with regard to the coevolution of the brain and eye. During ontogenetic development, the retina, an extension of the central nervous system, may prompt animals with larger brains to develop larger eyes to acquire more visual information from the environment [13,47,48]. Additionally, vision has been shown to be closely related to the structures of various brain regions [49]. Therefore, it is expected that the neural pathways and connections associated with visual information processing will increase as larger eyes become important sensory organs of a species, leading to the evolution of brain structures that improve adaptive behaviour in ecological environments [40,49,50,51]. This coevolution of the brain and eye has been found to improve adaptive behaviour performance in various species (e.g., amphibians [13] and birds [40]). However, a recent study has revealed that the relationship between eye size and overall brain size may not be consistent across all species, such as in the case of Trinidadian killifish (*Anablepsoides hartii*) where eye size was not found to be correlated with brain size, despite a positive connection with the evolution of brain structure [49].

In the present study, we aimed to investigate the coevolutionary relationships between eye size with ecological factors (e.g., habitat openness, food type and foraging habitat), behaviours (e.g., migration and activity pattern) and brain size in 1274 avian species using a phylogenetic comparative approach. We predict that: (i) birds living in dense habitats or with nocturnal habits will have larger eyes to cope with low light and complex environments; (ii) birds that are carnivorous or forage on the wing will have larger eyes for efficient tracking and capturing prey; (iii) larger-brained birds will develop more visual nerves, leading to larger eye size. Finally, we expect that (iv) migrant birds may not have the extra energy to develop larger eye sizes due to their energy demands during migration.

## 2. Materials and Methods

### 2.1. Data Collection

Data on eye morphology (axial length and transverse diameter) were obtained from published literature (Tables S1 and S2, Supplementary Materials). From this, we calculated eye volume using the equation: eye size (cm^3^) = 2 × 1.33πa^2^b, where a is half of the transverse diameter and b is half of the axial length, measured in cm [40,52]. In addition to eye volume, we also focused on the axial length as it is considered an important indicator of visual acuity at high speeds and the ability to resolve discrete objects [1,9,39,53]. Thus, we also extended the data on the axial length that included raptor species (Accipitriformes, Cathartiformes, Falconiformes and Strigiformes). Our dataset comprised data on the eye volumes for 1041 avian species and the axial lengths of 1274 avian species.

We also obtained data on absolute brain volume and body mass for 1274 avian species from the published literature (Tables S1 and S2, Supplementary Materials). To examine the coevolutionary relationship between eye size and brain size, we converted absolute brain mass from the literature to absolute brain volume (1.036 g/mL [54]) and calculated the relative brain size. Then, we augmented the dataset by extracting data on ecological factors (e.g., habitat openness, food type and foraging habitat) and behaviours (e.g., migration and activity pattern) for each species. According to the methods of Tobias et al. [55], habitat openness was classified into three categories, namely dense, semiopen and open. Migration was grouped into three categories (sedentary, partially migratory and migration), food type was categorized as plants, animals, omnivorous or carrion/refuse. Foraging habitat was classified as aerial, aquatic, terrestrial, insessorial or generalist (see Supplemental Methods in the Supplementary Materials for details of each category). Activity pattern was divided into two categories: diurnal and nocturnal. The final dataset included 1274 species, and the sample size varied for different variables (Tables S1 and S2, Supplementary Materials).

### 2.2. Phylogeny

**Figure 2:**
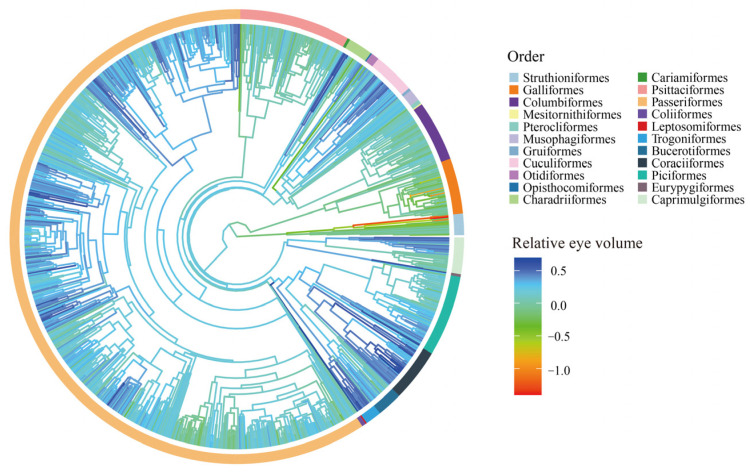
Distribution of relative eye volume across 1041 bird species. Log–log PGLS regression of absolute eye volume on body mass the ‘phyl.resid’ function in the R package ‘phytools’ was performed to calculate relative eye volume.

The phylogenetic information of 1274 bird species was obtained from birdtree.org [56]. Two types of phylogenetic trees with different numbers of species were used in our analysis, one for eye volume and another for axial length. We constructed the maximum clade credibility (MCC) tree (Figure 2) using TreeAnnotator, a program included in the software BEAST v1.10.4 [57], as the main phylogenetic hypothesis based on 10,000 phylogenetic trees. To account for phylogenetic uncertainty, we randomly extracted 50 phylogenetic trees that re-ran the key analyses. The 50 trees were defined as the most-efficient number required for our analysis [58,59].

### 2.3. Data Analysis

All analyses used statistical software R version 4.2.1 [60]. Continuous variables were log10-transformed for normalization before analysis. Body mass was controlled in all analyses because the sizes of eyes in vertebrates are proportional to body size [14,61]. Thus, the relative eye volume and relative axial length are the response variables in our analysis. To test the hypothesis between relative eye size and relative brain size, we implemented phylogenetic generalized least squares (PGLS) models [62] in the R package ‘ape’ [63]. The phylogenetic scaling parameter *λ* (0  ≤  *λ*  ≤  1) was estimated by the maximum likelihood approach [64], where *λ* = 0 means phylogenetic independence while to *λ* = 1 means complete phylogenetic dependence [62]. We first estimated the relative brain size via the ‘phyl.resid’ function in the R package ‘phytools’ [65], which avoids the strong correlation between absolute brain volume and body mass (*λ* = 0.906^<0.001,<0.001^, *R*^2^ = 0.975, *t* = 98.146, *p* < 0.001). The relative brain size is a good proxy for general domain cognition [66,67]. We used PGLS models treating eye volume or axial length as a response variable, relative brain size as a predictor variable and body mass as a covariate to test the effect of relative brain size on relative eye size evolution. *R*^2^ values (*R*^2^_lik_) were calculated in the R package ‘rr2’ [68].

To examine the effects of ecological factors and behaviours on relative eye size, we implemented Markov Chain Monte Carlo general linear mixed models in the R package ‘MCMCglmm’ [69]. The inverse-Wishart priors (V = 1, ν = 0.002) were used in all models. The MCMC chains were run for 210,000 iterations with a 10,000 burn-in and a thinning interval of 50. Eye volume or axial length as a dependent variable, with body mass as a covariate and habitat openness, migration, food type, foraging habitat or activity pattern as a fixed effect, respectively (e.g., the formula used for analysis: eye volume~habitat openness + body mass). In addition, we attempted to use the ratio (axial length/transverse diameter) as the dependent variable, but it was not associated with ecological factors and behaviours (Table S5).

The multipredictor models were developed based on important predictors in all bivariate models. We constructed a multipredictor model in MCMCglmm for the eye volume as the dependent variable, with relative brain size, habitat openness and food type as predictors and body mass as a covariate (Table S6). Then, the second multipredictor model contained axial length as the dependent variable and body mass as a covariate, with the following predictors: relative brain size, habitat openness, food type and activity pattern (Table S7). Two multipredictor models were examined in the R package ‘coda’ [70]. We ran each multipredictor model three times and tested Gelman and Rubin’s convergence diagnostic via the ‘gelman.diag’ function, in which the upper limits should be near 1. Trace plots were examined to ensure that there were no trends (Figures S1 and S2). We also tested Geweke’s convergence diagnostics via the ‘geweke.diag’ function, which indicated good convergence with all values less than the absolute value of 1.96 (Table S8). Additionally, density plots should display normality and symmetry (Figures S3 and S4 [70,71]). Eventually, we ran each multipredictor model 50 times using 50 phylogenetic trees to account for phylogenetic uncertainty. All variance inflation factors (VIF) were less than 1.182 in all multipredictor models.

## 3. Results

**Figure 3:**
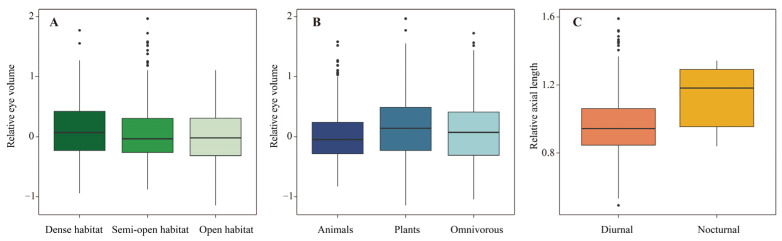
The boxplots for the relationship between eye volume (relative to body mass) with (**A**) habitat openness and (**B**) food type, and the association of axial length (relative to body mass) with (**C**) activity pattern of birds. Black points indicate the outliers.

The eye volume in our sample of 1041 bird species ranged between 0.072 cm^3^ (Vervain hummingbird, *Mellisuga minima*) and 92.807 cm^3^ (Common ostrich, *Struthio camelus*) with a mean of 2.334 cm^3^. Bivariate models in MCMCglmms showed that relative eye volume correlates with the environment (Table S3). Specifically, the species living in the dense habitat had relatively larger eye volumes than those living in semiopen (Posterior mean = −0.031, 95% CI: −0.048, −0.012, *P_mcmc_* < 0.001) or open habitats (posterior mean = −0.063, 95% CI: −0.089, −0.037, *P_mcmc_* < 0.001; Figure 3A). Moreover, animal-eating species had relatively larger eye volumes than plant-eating (posterior mean = −0.081, 95% CI: −0.114, −0.049, *P_mcmc_* < 0.001) or omnivorous species (posterior mean = −0.039, 95% CI: −0.067, −0.011, *P_mcmc_* = 0.006; Figure 3B). Migration did not predict relative eye volume (Table S3), as the relative eye volume and relative axial length were not different among sedentary, partially migratory and migratory species. Foraging habitat and activity pattern did not influence the relative eye volume of birds (Table S3).

The average axial length of all the 1274 species was 10.892 mm (range: 3.100 mm–38.925 mm). Consistent with our findings for relative eye volume, a greater relative axial length was significantly associated with habitat openness and feeding habitat, but not migration (Table S4). Nocturnal species possessed relatively longer axial lengths than diurnal species (posterior mean = 0.035, 95% CI: 0.006, 0.067, *P_mcmc_* = 0.022; Figure 3C). Meanwhile, aerial foragers possess relatively longer axial lengths than species foraging in water (posterior mean = −0.060, 95% CI: −0.110, −0.009, *P_mcmc_* = 0.024) or on the ground (posterior mean = −0.022, 95% CI: −0.040, −0.001, *P_mcmc_* = 0.032).

Phylogenetic generalized least-squares models revealed that relative eye volume (*N_species_* = 1041, *R*^2^ = 0.922, *t* = 6.582, *p* < 0.001, *λ* = 0.863^<0.001,<0.001^) and relative axial length (*N_species_* = 1274, *R*^2^ = 0.920, *t* = 8.416, *p* < 0.001, *λ* = 0.853^<0.001,<0.001^) were both positively correlated with relative brain size. Using multipredictor models in MCMCglmm, we also found that relative eye volume was significantly associated with relative brain size, habitat openness and food type (Table 1 and Table S6).

Similarly, using multipredictor models in MCMCglmm, the relative axial length was also associated with relative brain size, habitat density and trophic level, but not with activity pattern (Table 2 and Table S7).

## 4. Discussion

Our study found that species with larger eye sizes are more likely to inhabit dense habitats, consume invertebrates or vertebrates and tend to possess larger brains. Additionally, our analyses indicated that foraging habitat and activity patterns may have affected the evolution of avian eye size, with species foraging in aerial environments having longer axial lengths compared to those feeding in aquatic and terrestrial environments and nocturnal birds possessing longer axial lengths than diurnal ones. These results suggest that habitat openness and food type play critical roles in shaping visual sensitivity and visual acuity, thereby influencing the evolution of avian eye size.

The requirements for visual sensitivity and visual acuity in dim light are significant determinants of eye size since the eyes are susceptible to their surroundings [50,72]. In the study, we found that birds living in dense habitats tended to have relatively larger eye sizes than those living in semiopen habitats, highlighting the complexity and light levels of habitats have a significant impact on eye size in animals. Our findings are consistent with previous studies that have shown that animals living in dimly lit or arboreal habitats, such as reef fishes, tree frogs and forest birds, tend to have larger eyes for better visual sensitivity [7,25,28,73]. However, some vertebrates that live in dark habitats, such as mole rats, eels, fossorial lizards and kiwi, have reduced eye size over time and associated neurological structures are also reduced [74,75,76,77]. In addition, prior studies have shown that the distribution of avian retinal ganglion cells is closely linked to habitat openness, which conforms to the “terrain” theory [78]. Specifically, the retinas of birds living in open areas are specialized and have a well-defined visual streak, which is a horizontally elongated area of increased cell density in the retina [27,79,80,81]. Species living in open habitats and forests have cone visual streaks, allowing panoramic views without the need for larger eyes or head movements [78,82,83].

We also found that activity pattern affects relative axial length among avian species, with nocturnal species displaying longer relative axial lengths, presumably to resolve discrete objects in low-light conditions. A longer eye allows for greater visual acuity and lower visual sensitivity [15]. However, nocturnal species may experience a disadvantage due to the resulting reduction in visual sensitivity. Nonetheless, a larger corneal diameter may serve as an advantage to offset this challenge [15]. Furthermore, the relative eye volume of birds is positively and significantly affected by nocturnal species that capture moving prey and not for all nocturnal species [40]. However, we did not observe any significant association between activity pattern and relative eye volume in our sample of 1041 avian species, which may be due to the limited number of nocturnal species included in our analyses.

Avian visual capabilities are crucial for their foraging ecology, and differences in eye size across species are evidence of their importance in this context. Larger eyes may be beneficial for the detection of prey, consistent with findings that predatory species have finer vision acuity than nonpredatory species in insects [84], elasmobranchs [85], mammals [86] and birds [40]. Additionally, large eyes are also beneficial for the long-distance detection of prey in birds [14,25]. Our study revealed that birds foraging on invertebrates or vertebrates had larger eyes than birds foraging on plants, carrion or refuse, or omnivores. Carnivorous birds need higher vision to capture food, thereby reducing excess energy consumption during predation. This suggests that variations in avian eye size may be driven by the need to capture dynamic food and that food type can be a significant factor influencing eye evolution.

Notably, in addition to the food type that birds forage on, different foraging environments can also affect the evolution of eyes in animal groups. Ray-finned fishes that forage in clear water have high eye investments to compensate for the attenuation of light in water, which is quicker than in air [73]. Moreover, fully aquatic anurans are likely to have smaller eye sizes than semiaquatic anurans because they forage in highly turbid environments [7]. Inconsistent with our prediction, we found that the foraging habitat cannot explain eye size evolution across 1274 species of birds, despite differences in axial length in species foraging in aerial habitats compared to other foraging habitats. We speculate that avian eyes adapt to foraging habitats through alternative mechanisms, such as the ability of Ardeidae to improve their visual fields by changing the skull’s eye position [32]. Moreover, some bird species, such as ducks and flamingos, which forage through filter feeding, do not require much visual information, and may not exhibit eye size evolution facilitated by foraging habitat [32].

The environmental information projects to the retinal and are then mainly received by the optic tectum, which, in turn, projects to the nucleus rotundus and finally to the telencephalon [87,88,89]. Interestingly, eye size and brain size are known to be codevelopmentally linked in animal groups [90]. This relationship may be driven by the evolutionary need to enhance fitness in ecological environments, thereby necessitating the development of larger eyes and brain structures [13,40,49,50]. This relationship between eye and brain size appears to be widespread among vertebrates including amphibians and birds [13,40,50]. However, space available in the skull also determines the eye size of birds because eye size is proportionally much larger compared to the given body size [14,50,61]. Moreover, the ratio of eyes to brain is often isometric in nonpasserine birds, with a larger relative brain size being associated with a larger relative eye size [50]. Our analysis is consistent with these previous findings and suggests that the evolution of the eye and brain in birds is a coadaptive process driven by the need to process visual information in complex and changing environments.

The energetic cost of maintaining larger eyes is an important consideration in understanding the evolution of eye size in birds [7,45,46]. The flight speed of birds may exert negative selection pressure on the evolution of eye size. In a previous study of 88 bird species, migration speeds can negatively affect eye size evolution, with faster-flying birds exhibiting smaller eye sizes, potentially due to the negative selection pressure imposed by migration [16]. Interestingly, in nonforest species, migratory birds exhibit smaller eye sizes, which may reflect their adaptation to a wider range of light levels and larger environments [25]. However, in our analysis of 1274 bird species, we did not find a significant negative relationship between migration behaviour and eye size evolution. It is possible that compensatory behaviours, such as rest and sleep, mitigate the energetic costs of migration [91]. Additionally, nocturnal migration may promote the development of larger eyes adapted to low-light levels [91,92,93]. Hence, the influence of migration on eye size evolution is complex and requires further investigation.

## 5. Conclusions

Birds have been widely studied due to their unique importance of vision in the environment, and they possess relatively large eyes compared to other terrestrial vertebrates [9,14,15,40,94]. In this study, we first used eye volume and axial length as indicators of eye size to examine the coevolutionary relationship between eye size with ecological factors, behaviours and brain size in a sample of 1274 birds, indicating the importance of light levels in shaping avian eye size evolution. Furthermore, food type is also associated with the evolution of avian eye size, suggesting that a strong foraging need can promote a wider range of visual fields. A positive relationship between relative eye size and relative brain size has suggested that enlarged eyes require larger brains for processing environmental information. In addition, further studies incorporating more detailed data on foraging and flight are needed to advance comparative phylogenetic analyses in this field.

## Figures and Tables

**Table 1:** Phylogenetically controlled mixed models in MCMCglmm assessing the effect of relative brain size, habitat openness and food type on avian relative eye volume (n = 1041 species). Model averaging results of 50 Bayesian phylogenetic mixed models.

Variables	Posterior Mean	Lower 95% CI	Upper 95% CI	*P_mcmc_*
(Intercept)	−1.347	−1.510	−1.182	<0.001
Relative brain size	0.342	0.227	0.455	**<0.001**
Habitat openness (dense habitat vs. open habitat)	−0.057	−0.083	−0.031	**<0.001**
Habitat openness (dense habitat vs. semi-open habitat)	−0.025	−0.043	−0.007	**0.007**
Food type (animals vs. plants)	−0.076	−0.108	−0.045	**<0.001**
Food type (animals vs. omnivorous)	−0.037	−0.064	−0.010	**0.009**
Body mass	0.725	0.700	0.750	<0.001

Significant predictors are marked in bold.

**Table 2:** Phylogenetically controlled mixed models in MCMCglmm assessing the effect of relative brain size, ecological factors and behaviours on avian relative axial length (n = 1274 species). Model averaging results of 50 Bayesian phylogenetic mixed models.

Variables	Posterior Mean	Lower 95% CI	Upper 95% CI	*P_mcmc_*
(Intercept)	0.496	0.433	0.559	<0.001
Relative brain size	0.155	0.114	0.195	**<0.001**
Habitat openness (dense habitat vs. open habitat)	−0.019	−0.028	−0.010	**<0.001**
Habitat openness (dense habitat vs. semiopen habitat)	−0.008	−0.014	−0.001	**0.024**
Food type (animals vs. plants)	−0.028	−0.039	−0.016	**<0.001**
Food type (animals vs. omnivorous)	−0.014	−0.024	−0.003	**0.009**
Food type (animals vs. carrion or refuse)	−0.064	−0.120	−0.008	**0.031**
Activity pattern (diurnal vs. nocturnal)	0.020	−0.010	0.049	0.206
Body mass	0.236	0.227	0.245	<0.001

Significant predictors are marked in bold.

## Data Availability

The data presented in this study are available in the article and Supplementary Materials.

## Supplementary Materials

The following supporting information can be downloaded at: https://www.mdpi.com/article/10.3390/ani13101675/s1, Table S1: Data of axial length, transverse diameter, brain volume, body mass, habitat openness, migration, food type, foraging habitat and activity pattern used for this study. Habitat openness was classified into three categories: De = dense habitat, So = semiopen habitat and Op = open habitat. Migration was classified into three categories: Se = sedentary, Pm = partially migratory and Mi = migratory. Food type was classified into four categories: Pl = plants, An = animals, Om = omnivorous, Ca/Re = carrion or refuse. Foraging habitat was classified into five categories: Ae = aerial, Te = terrestrial, In = insessorial, Aq = aquatic and Ge = generalist. Activity pattern was classified into two categories: D = diurnal and N = nocturnal; Table S2: Details of species traits and the number of species sampled in our dataset. References are listed at the end of this document; Table S3: Five separate MCMCglmm models to test for interactions between relative eye volume with either ecological or behavioural variables (e.g., eye volume∼habitat openness + body mass). Significant predictors are marked in bold; Table S4: Five separate MCMCglmm models to test for interactions between relative axial length with either ecological or behavioural variables (e.g., axial length∼habitat openness + body mass). Significant predictors are marked in bold; Table S5: Phylogenetically controlled mixed models in MCMCglmm assessing the effect of ecological factors and behaviours on the ratio (axial length/transverse diameter) in 1041 species of birds; Table S6: Phylogenetically controlled mixed models in MCMCglmm assessing the effect of relative brain size and ecological variables on relative eye volume in 1041 species of birds. Significant predictors are marked in bold; Table S7: Phylogenetically controlled mixed models in MCMCglmm assessing the effect of relative brain size and ecological and behavioural variables on relative axial length in 1274 species of birds. Significant predictors are marked in bold; Table S8: Diagnostic values for assessing our two multipredictor models for MCMCglmm analyses. Values for (a) eye volume~relative brain size + habitat openness + food type + body mass, and (b) axial length~relative brain size + habitat openness + food type + activity pattern + body mass; Figure S1: Trace plots for fixed factors in the first multipredictor model (eye volume~relative brain size + habitat openness + food type + body mass). See Table S8 for additional diagnostics; Figure S2: Trace plots for fixed factors in the second multipredictor model (axial length~relative brain size + habitat openness + food type + activity pattern + body mass). See Table S8 for additional diagnostics; Figure S3: Density plots for fixed factors in the first multipredictor model (eye volume~relative brain size + habitat openness + food type + body mass). See Table S8 for additional diagnostics; Figure S4: Density plots for fixed factors in the second multipredictor model (axial length~relative brain size + habitat openness + food type + activity pattern + body mass). See Table S8 for additional diagnostics. Refs. [95,96,97,98,99,100,101,102,103,104,105,106,107,108,109,110,111] are cited in the Supplementary Materials.
