# Supplementary material for: Evolution of Avian Eye Size Is Associated with Habitat Openness, Food Type and Brain Size
Source: Animals (Basel). 2023 May 18;13(10):1675. doi: 10.3390/ani13101675 (PMC10215482; doi:10.3390/ani13101675)
Supplement: Supplementary file 1 [file animals-13-01675-s001.zip › animals-2358937-supplementary.pdf]

Evolution of avian eye size is associated with habitat openness, food type and brain size

Yating Liu [1], Ying Jiang [1], Jiliang Xu [1,*] and Wenbo Liao [2,3,*]

[1]School of Ecology and Nature Conservation, Beijing Forestry University, Beijing 100083, China

[2]Key Laboratory of Southwest China Wildlife Resources Conservation (Ministry of Education), China West Normal University, Nanchong 637009, China

[3]Key Laboratory of Artificial Propagation and Utilization in Anurans of Nanchong City, China West Normal University, Nanchong 637009, China

*Correspondence: xujiliang@bjfu.edu.cn (J.X.); liaobo_0_0@126.com (W.L.)

SUPPLEMENTARY MATERIAL

Supplemental Methods

Tables S1 to S8

Figures S1 to S4

Supplemental Methods

Habitat openness was categorized into dense habitat: species primarily live in a dense environment with not too much light (e.g., the lower storey of forest or dense thickets); semiopen habitat: species primarily live in the environment with more light (e.g., open shrubland or parkland); and open habitat: species primarily lives in the open environment with abundant light and wide vision (e.g., desert or grassland).

Migration of bird species was categorized as follows: sedentary: species that live in breeding grounds and do not migrate, such as magpie and short tailed parrot; partially migratory: species that short distance migration, nomadic movements, distinct altitudinal migration, or part of population migration, such as fairy martin and common buzzard; and migratory: species that migrate long distances on periodic, such as common cuckoo and white stork.

Food type of bird species included four categories: plants, animals (invertebrate or vertebrate), carrion or refuse and omnivorous. Except for 'omnivorous', other categories were allocated based on the mainly eaten food category (at least 70%). Then, 'omnivorous' was defined as food in roughly equal proportions from plants and animals.

Foraging habitat was classified into five categories: aerial: species foraging in flight and hunting on the wings (e.g., long−eared owl and golden eagle); terrestrial: species foraging on the ground by walk or hop (e.g., pheasant and water pipit); insessorial: species perching above the substrates (e.g., ground, arboreal, rocks and wires) before foraging (e.g., laughing kookaburra and wreathed hornbill); aquatic:

species foraging sit on the water by afloat or diving (e.g., whooper swan and penguin); and generalist: species spends time in different foraging habitat classes (e.g., tawny eagle and red avadavat).

Table S1 Data of axial length, transverse diameter, brain volume, body mass, habitat openness, migration, food type, foraging habitat and activity pattern used for this study. Habitat openness was classified into three categories: De = dense habitat, So = semiopen habitat, Op = open habitat. Migration was classified into three categories: Se = sedentary, Pm = partially migratory, Mi = migratory. Food type was classified into four categories: Pl = plants, An = animals, Om = omnivorous, Ca/Re = carrion or refuse. Foraging habitat was classified into five categories: Ae = aerial, Te = terrestrial, In = insessorial, Aq = aquatic, Ge = generalist. Activity pattern was classified into two categories: D = diurnal, N = nocturnal.

Species	Order	Axial length (mm)	Transverse diameter (mm)	Brain volume (ml)	Body mass (g)	Habitat openness	Migration	Food type	Foraging habitat	Activity pattern
Abroscopus_albogularis	Passeriformes	5.00	6.30	0.32	4.84	De	Se	An	In	D
Acanthagenys_rufogularis	Passeriformes	9.00	10.55	1.16	47.80	So	Se	Om	In	D
Acanthisitta_chloris	Passeriformes	5.85	6.64	0.33	6.91	De	Se	An	In	D
Acanthiza_chrysorrhoa	Passeriformes	5.50	6.65	0.43	9.29	So	Se	An	Te	D
Acanthiza_pusilla	Passeriformes	6.40	7.35	0.45	7.62	So	Se	An	In	D
Acanthorhynchus_superciliosus	Passeriformes	4.80	6.30	0.79	9.72	So	Se	Pl	In	D
Acanthorhynchus_tenuirostris	Passeriformes	5.90	7.20	0.48	10.44	So	Se	Om	In	D
Accipiter_badius	Accipitriformes	15.40	NA	2.83	131.15	So	Mi	An	In	D
Accipiter_cooperii	Accipitriformes	18.00	NA	4.70	429.67	So	Mi	An	Ae	D
Accipiter_gentilis	Accipitriformes	22.30	NA	7.88	866.04	De	Pm	An	Ge	D
Accipiter_nisus	Accipitriformes	13.50	NA	2.85	220.79	De	Pm	An	In	D
Accipiter_novaehollandiae	Accipitriformes	17.80	NA	4.74	326.06	De	Se	An	In	D
Accipiter_striatus	Accipitriformes	13.40	NA	2.23	130.59	De	Mi	An	Ae	D
Accipiter_tachiro	Accipitriformes	16.80	NA	4.15	290.20	De	Se	An	Ae	D
Aceros_plicatus	Bucerotiformes	20.60	23.98	15.42	1703.76	De	Se	Pl	In	D
Aceros_undulatus	Bucerotiformes	20.80	24.40	18.25	2214.55	De	Se	Pl	In	D
Acridotheres_tristis	Passeriformes	10.70	12.85	2.48	116.43	Op	Pm	An	Te	D
Acrocephalus_schoenobaenus	Passeriformes	5.80	7.25	0.45	11.83	Op	Mi	An	In	D
Acrocephalus_scirpaceus	Passeriformes	6.30	7.65	0.46	12.30	Op	Mi	An	In	D
Acrocephalus_stentoreus	Passeriformes	6.90	8.70	0.84	24.20	Op	Pm	An	In	D
Acryllium_vulturinum	Galliformes	15.30	18.00	4.55	1330.00	So	Se	Pl	Te	D
Actitis_hypoleucos	Charadriiformes	7.40	NA	0.86	48.00	Op	Mi	An	Te	D

Actitis_macularius	Charadriiformes	7.30	NA	0.68	40.40	Op	Mi	An	Te	D
Actophilornis_africanus	Charadriiformes	10.40	NA	0.79	189.09	Op	Pm	An	Te	D
Aegithalos_caudatus	Passeriformes	6.30	6.95	0.43	8.60	So	Se	An	In	D
Aegithalos_concinnus	Passeriformes	4.90	6.28	0.37	6.10	De	Se	An	In	D
Aegithina_tiphia	Passeriformes	6.20	7.85	0.67	12.00	De	Se	An	In	D
Aegolius_acadicus	Strigiformes	15.65	NA	3.36	100.69	So	Mi	An	In	N
Aegolius_funereus	Strigiformes	17.00	NA	4.02	137.18	De	Pm	An	In	N
Aegotheles_cristatus	Caprimulgiformes	13.17	NA	1.42	42.78	So	Se	An	In	N
Aeronautes_saxatalis	Caprimulgiformes	9.10	11.45	0.67	32.10	De	Se	An	Ae	D
Aethopyga_nipalensis	Passeriformes	5.05	6.58	0.33	6.90	De	Se	Om	In	D
Agapornis_canus	Psittaciformes	5.50	7.20	1.15	30.00	So	Se	Pl	In	D
Agapornis_fischeri	Psittaciformes	7.55	8.85	1.95	48.30	Op	Se	Pl	In	D
Agelaius_phoeniceus	Passeriformes	7.14	7.53	1.69	50.78	Op	Mi	Pl	Ge	D
Ailuroedus_crassirostris	Passeriformes	13.30	17.45	4.01	205.32	De	Se	Pl	In	D
Alcedo_atthis	Coraciiformes	8.30	10.18	0.74	31.09	So	Se	An	In	D
Alcedo_azurea	Coraciiformes	8.80	10.85	0.98	34.90	So	Se	An	In	D
Alcedo_cristata	Coraciiformes	7.30	8.80	0.54	15.70	So	Se	An	In	D
Alcippe_nipalensis	Passeriformes	7.50	8.65	0.93	15.80	De	Se	Om	In	D
Alectoris_chukar	Galliformes	13.50	15.10	2.48	502.10	Op	Se	Pl	Te	D
Alectura_lathami	Galliformes	16.00	21.10	5.68	2333.06	De	Se	Pl	Te	D
Alethe_diademata	Passeriformes	9.60	10.95	1.04	31.54	De	Se	An	Ge	D
Alisterus_scapularis	Psittaciformes	12.40	14.30	4.48	232.26	De	Se	Pl	In	D
Alle_alle	Charadriiformes	10.49	NA	2.10	180.73	Op	Mi	An	Aq	D
Amadina_fasciata	Passeriformes	5.30	6.45	0.59	18.00	Op	Se	Pl	Te	D
Amalocichla_incerta	Passeriformes	8.00	11.35	1.10	32.70	De	Se	An	Te	D
Amandava_amandava	Passeriformes	4.40	5.50	0.38	8.97	So	Se	Pl	Ge	D
Amazilia_tzacatl	Caprimulgiformes	4.13	NA	0.22	4.79	De	Se	Pl	Ae	D
Amazona_albifrons	Psittaciformes	12.20	14.20	5.28	206.00	So	Se	Pl	In	D
Amazona_ochrocephala	Psittaciformes	15.30	17.65	8.64	476.94	So	Se	Pl	In	D
Amblycercus_holosericeus	Passeriformes	8.50	10.45	2.21	70.42	De	Se	An	In	D
Amblyornis_macgregoriae	Passeriformes	13.10	15.85	3.58	117.47	De	Se	Pl	In	D
Amblyornis_subalaris	Passeriformes	12.60	15.20	3.55	108.88	De	Se	Om	In	D

Amblyramphus_holosericeus	Passeriformes	8.30	9.70	2.33	56.76	So	Se	An	In	D
Amphispiza_bilineata	Passeriformes	6.50	7.65	0.68	13.50	So	Pm	Om	Ge	D
Anas_acuta	Anseriformes	9.99	NA	4.72	944.62	Op	Mi	Pl	Aq	D
Anas_crecca	Anseriformes	10.45	NA	2.70	341.89	Op	Pm	Pl	Aq	D
Anas_platyrhynchos	Anseriformes	13.20	NA	5.44	843.42	Op	Pm	Pl	Aq	D
Andigena_hypoglauca	Piciformes	16.30	19.40	4.65	312.83	De	Se	Pl	In	D
Anisognathus_somptuosus	Passeriformes	8.40	9.85	1.49	44.50	De	Se	Om	In	D
Anodorhynchus_hyacinthinus	Psittaciformes	16.80	19.80	24.73	1331.00	So	Se	Pl	In	D
Anorrhinus_galeritus	Bucerotiformes	21.80	23.85	9.60	1172.00	De	Se	Om	In	D
Anser_albifrons	Anseriformes	14.80	NA	9.09	2506.39	Op	Mi	Pl	Te	D
Anthracoceros_coronatus	Bucerotiformes	19.30	22.45	7.98	806.72	So	Se	Pl	In	D
Anthracothorax_dominicus	Caprimulgiformes	4.50	5.90	0.23	5.77	So	Se	Pl	Ae	D
Anthus_berthelotii	Passeriformes	7.30	8.75	0.50	15.86	So	Se	An	Te	D
Anthus_campestris	Passeriformes	7.80	9.40	0.46	23.00	Op	Mi	An	Te	D
Anthus_novaeseelandiae	Passeriformes	7.80	9.35	0.84	24.07	Op	Se	An	Te	D
Anthus_spinoletta	Passeriformes	6.30	8.25	0.75	23.90	Op	Mi	An	Te	D
Anthus_trivialis	Passeriformes	7.40	8.50	0.60	23.33	So	Mi	Om	Te	D
Anumbius_annumbi	Passeriformes	8.85	10.18	1.18	41.50	Op	Se	An	Ge	D
Apaloderma_narina	Trogoniformes	11.90	13.90	1.52	67.69	De	Se	An	In	D
Aphelocephala_nigricincta	Passeriformes	6.20	6.85	0.47	10.40	So	Se	An	Te	D
Aphelocoma_coerulescens	Passeriformes	11.50	14.05	2.85	77.12	So	Se	Om	In	D
Aphelocoma_ultramarina	Passeriformes	12.30	15.40	3.61	113.33	So	Se	Om	Ge	D
Aphrastura_spinicauda	Passeriformes	6.70	7.75	0.81	11.50	So	Se	An	In	D
Aplonis_atrifusca	Passeriformes	11.00	12.95	2.29	146.00	So	Se	Pl	In	D
Aplonis_panayensis	Passeriformes	9.40	11.00	1.39	56.20	De	Se	Pl	In	D
Aprosmictus_erythropterus	Psittaciformes	8.78	10.49	3.85	136.00	So	Se	Pl	In	D
Apteryx_australis	Struthioniformes	6.91	7.44	10.86	2320.51	De	Se	An	Te	N
Apteryx_owenii	Struthioniformes	7.30	7.40	7.30	1238.29	De	Se	An	Te	N
Apus_affinis	Caprimulgiformes	6.79	7.95	0.52	22.57	De	Mi	An	Ae	D
Apus_apus	Caprimulgiformes	9.80	11.75	0.67	37.60	De	Mi	An	Ae	D
Apus_pallidus	Caprimulgiformes	10.00	12.15	0.72	41.90	De	Mi	An	Ae	D
Aquila_chrysaetos	Accipitriformes	29.55	NA	17.19	4247.97	Op	Pm	An	Ae	D

Aquila_pomarina	Accipitriformes	26.80	NA	10.91	1359.41	De	Mi	An	Ge	D
Aquila_rapax	Accipitriformes	27.80	NA	13.31	2236.06	So	Mi	An	Ge	D
Ara_ararauna	Psittaciformes	15.80	19.05	18.08	1125.00	De	Se	Pl	In	D
Ara_militaris	Psittaciformes	16.00	19.25	18.83	1134.00	So	Se	Pl	In	D
Ara_severus	Psittaciformes	12.00	16.50	10.05	343.00	De	Se	Pl	In	D
Arachnothera_longirostra	Passeriformes	6.00	7.70	0.52	12.46	De	Se	Om	In	D
Aratinga_leucophthalma	Psittaciformes	10.90	13.15	5.58	158.00	So	Se	Pl	In	D
Aratinga_pertinax	Psittaciformes	9.40	10.90	3.49	83.99	So	Se	Pl	In	D
Aratinga_weddellii	Psittaciformes	8.59	NA	4.01	108.00	De	Se	Pl	In	D
Archilochus_colubris	Caprimulgiformes	3.93	4.70	0.15	3.09	So	Mi	Om	Ge	D
Ardea_cinerea	Pelecaniformes	18.70	NA	7.85	1443.00	Op	Se	An	Te	D
Ardeotis_australis	Otidiformes	28.60	35.10	11.65	4182.35	Op	Se	Om	Te	D
Ardeotis_kori	Otidiformes	33.15	39.13	12.93	7994.16	Op	Se	Om	Te	D
Arenaria_interpres	Charadriiformes	9.70	NA	1.34	135.98	Op	Mi	An	Te	D
Arenaria_melanocephala	Charadriiformes	8.57	NA	1.53	126.21	Op	Mi	An	Te	D
Argusianus_argus	Galliformes	18.40	22.10	5.68	1959.93	De	Se	Pl	Te	D
Arremonops_conirostris	Passeriformes	8.40	10.00	1.29	35.89	So	Se	Om	Te	D
Arses_telescophthalmus	Passeriformes	7.70	8.90	0.62	15.26	De	Se	An	In	D
Artamus_cinereus	Passeriformes	8.83	10.28	0.92	35.60	So	Se	An	Ae	D
Artamus_cyanopterus	Passeriformes	8.30	9.75	1.00	34.60	So	Se	An	Ae	D
Artamus_leucorynchus	Passeriformes	9.90	11.85	1.08	41.69	So	Se	An	Ae	D
Artamus_maximus	Passeriformes	9.80	12.30	1.65	58.20	So	Se	An	Ae	D
Artamus_personatus	Passeriformes	9.00	10.30	1.03	34.60	So	Se	An	Ae	D
Artamus_superciliosus	Passeriformes	9.00	10.30	1.04	35.50	So	Mi	An	Ae	D
Ashbyia_lovensis	Passeriformes	7.00	8.95	0.67	17.50	Op	Pm	An	Te	D
Asio_flammeus	Strigiformes	19.00	NA	5.30	322.61	Op	Mi	An	Ae	D
Asio_otus	Strigiformes	17.50	NA	5.31	296.57	So	Pm	An	Ae	N
Athene_brama	Strigiformes	18.00	NA	3.34	112.00	So	Se	An	In	N
Athene_cunicularia	Strigiformes	18.50	NA	3.78	150.61	Op	Se	An	Ge	D
Athene_noctua	Strigiformes	18.40	NA	3.70	168.92	Op	Se	An	In	N
Attila_spadiceus	Passeriformes	11.00	12.70	1.18	39.10	De	Se	An	In	D
Aulacorhynchus_sulcatus	Piciformes	13.30	16.25	2.74	167.00	De	Se	Om	In	D

Auriparus_flaviceps	Passeriformes	5.10	6.25	0.44	6.80	So	Se	An	In	D
Automolus_infuscatus	Passeriformes	8.50	10.10	1.13	32.90	De	Se	An	In	D
Automolus_ochrolaemus	Passeriformes	8.40	9.80	1.14	40.20	De	Se	An	In	D
Aviceda_leuphotes	Accipitriformes	20.80	NA	3.28	193.98	So	Mi	An	In	D
Aviceda_subcristata	Accipitriformes	22.80	NA	4.70	322.65	So	Se	An	In	D
Baeolophus_bicolor	Passeriformes	7.70	8.80	1.01	21.60	De	Se	An	In	D
Balearica_pavonina	Gruiformes	18.93	NA	12.88	3590.00	Op	Se	Om	Te	D
Bambusicola_thoracicus	Galliformes	10.40	12.40	1.78	264.79	So	Se	Pl	Te	D
Barnardius_zonarius	Psittaciformes	10.50	12.75	3.91	133.00	So	Se	Pl	In	D
Bartramia_longicauda	Charadriiformes	11.20	NA	1.30	158.92	Op	Mi	An	Te	D
Baryphthengus_ruficapillus	Coraciiformes	15.50	18.90	2.65	141.65	De	Se	An	In	D
Basileuterus_culicivorus	Passeriformes	6.15	7.70	0.58	10.50	De	Se	An	In	D
Batis_capensis	Passeriformes	7.40	8.75	0.68	11.74	So	Se	An	In	D
Bias_musicus	Passeriformes	8.80	10.05	0.84	21.90	De	Pm	An	In	D
Bolbopsittacus_lunulatus	Psittaciformes	8.10	11.00	2.65	67.60	De	Se	Pl	In	D
Bolborhynchus_lineola	Psittaciformes	7.70	9.25	2.08	56.20	De	Pm	Pl	In	D
Bombycilla_cedrorum	Passeriformes	7.67	9.52	0.89	31.58	So	Mi	Pl	In	D
Bombycilla_garrulus	Passeriformes	8.97	10.67	1.16	54.41	De	Mi	Pl	In	D
Bonasa_bonasia	Galliformes	11.15	14.90	1.82	429.00	De	Se	Pl	Ge	D
Bonasa_umbellus	Galliformes	10.28	12.61	2.46	530.91	De	Se	Pl	Ge	D
Botaurus_stellaris	Pelecaniformes	13.80	NA	5.89	1319.45	De	Pm	An	Te	D
Brachygalba_lugubris	Piciformes	7.80	8.70	0.43	15.90	So	Se	An	In	D
Brotogeris_versicolurus	Psittaciformes	8.30	9.98	2.39	71.53	So	Se	Pl	In	D
Bubalornis_albirostris	Passeriformes	9.50	11.38	2.13	61.90	Op	Se	Om	Te	D
Bubo_bubo	Strigiformes	35.30	NA	17.09	2668.51	So	Se	An	In	N
Bubo_lacteus	Strigiformes	24.50	NA	16.89	2114.94	Op	Se	An	In	N
Bubo_poensis	Strigiformes	23.50	NA	8.69	654.94	De	Se	An	In	N
Bubo_virginianus	Strigiformes	34.55	NA	14.73	1354.50	So	Se	An	In	N
Bucephala_clangula	Anseriformes	10.80	NA	5.95	918.56	Op	Mi	An	Aq	D
Buceros_bicornis	Bucerotiformes	18.20	21.45	18.25	2790.79	De	Se	Om	In	D
Buceros_hydrocorax	Bucerotiformes	17.50	21.80	17.00	1409.59	De	Se	Pl	In	D
Bucorvus_abyssinicus	Bucerotiformes	29.30	32.90	23.75	4000.00	Op	Se	An	Te	D

Bucorvus_cafer	Bucerotiformes	30.60	34.55	26.15	3743.62	Op	Se	An	Te	D
Burhinus_bistriatus	Charadriiformes	22.00	25.95	4.66	787.00	Op	Se	An	Te	N
Burhinus_capensis	Charadriiformes	19.55	25.03	3.67	422.99	Op	Se	An	Te	N
Burhinus_oedicnemus	Charadriiformes	19.30	23.35	3.65	459.00	Op	Mi	An	Te	N
Burhinus_superciliaris	Charadriiformes	21.80	25.95	3.30	480.00	Op	Se	An	Te	N
Busarellus_nigricollis	Accipitriformes	20.80	NA	8.75	766.14	De	Se	An	In	D
Butastur_teesa	Accipitriformes	18.90	NA	4.73	325.00	Op	Se	An	In	D
Buteo_buteo	Accipitriformes	20.80	NA	7.94	759.10	Op	Pm	An	In	D
Buteo_galapagoensis	Accipitriformes	23.60	NA	8.30	1137.44	De	Se	An	Ae	D
Buteo_jamaicensis	Accipitriformes	22.80	NA	9.18	1101.16	So	Mi	An	In	D
Buteo_lagopus	Accipitriformes	27.10	NA	9.20	949.76	Op	Mi	An	In	D
Buteo_lineatus	Accipitriformes	23.05	NA	7.19	603.72	De	Mi	An	Ae	D
Buteo_magnirostris	Accipitriformes	19.90	NA	4.56	269.00	De	Se	An	In	D
Buteo_platypterus	Accipitriformes	20.25	NA	5.12	453.65	So	Mi	An	In	D
Buteo_rufofuscus	Accipitriformes	20.10	NA	9.17	1530.00	Op	Pm	An	Ge	D
Buteogallus_anthracinus	Accipitriformes	21.00	NA	7.17	975.09	So	Se	An	In	D
Buteogallus_urubitinga	Accipitriformes	23.50	NA	10.17	1152.87	So	Se	An	In	D
Bycanistes_cylindricus	Bucerotiformes	18.90	23.20	10.13	921.00	De	Se	Pl	In	D
Cacatua_alba	Psittaciformes	15.30	19.05	14.16	570.00	De	Se	Pl	In	D
Cacatua_ducorpsii	Psittaciformes	12.40	14.40	8.70	394.00	De	Se	Pl	In	D
Cacatua_galerita	Psittaciformes	14.00	17.15	14.24	720.43	De	Se	Pl	In	D
Cacatua_haematuropygia	Psittaciformes	12.60	15.20	8.50	288.00	So	Se	Pl	In	D
Cacatua_leadbeateri	Psittaciformes	12.20	15.20	8.47	396.83	So	Se	Pl	In	D
Cacatua_roseicapilla	Psittaciformes	12.10	12.90	6.43	328.00	Op	Se	Pl	Te	D
Cacatua_sanguinea	Psittaciformes	12.20	13.90	8.91	523.69	So	Se	Pl	Te	D
Cacicus_cela	Passeriformes	10.90	12.80	2.43	85.45	De	Se	Om	In	D
Cacomantis_castaneiventris	Cuculiformes	9.50	12.80	0.95	33.20	De	Se	An	In	D
Cacomantis_flabelliformis	Cuculiformes	11.00	13.40	1.09	49.80	So	Se	An	In	D
Cacomantis_variolosus	Cuculiformes	9.10	12.40	0.97	35.75	De	Se	An	In	D
Calamanthus_fuliginosus	Passeriformes	7.60	8.80	0.70	20.81	So	Se	An	Te	D
Calamospiza_melanocorys	Passeriformes	8.40	9.50	1.18	37.60	Op	Mi	Om	Ge	D
Calandrella_cinerea	Passeriformes	7.00	8.15	0.67	23.70	Op	Pm	Om	Te	D

Calidris_alba	Charadriiformes	7.10	NA	1.01	51.71	Op	Mi	An	Te	D
Calidris_alpina	Charadriiformes	6.70	NA	1.00	51.89	Op	Mi	An	Te	D
Calidris_bairdii	Charadriiformes	7.20	NA	0.73	40.97	Op	Mi	An	Te	D
Calidris_canutus	Charadriiformes	8.80	NA	1.32	141.87	Op	Mi	An	Te	D
Calidris_fuscicollis	Charadriiformes	6.60	NA	0.78	43.01	Op	Mi	An	Te	D
Calidris_mauri	Charadriiformes	6.00	NA	0.64	27.82	Op	Mi	An	Te	D
Calidris_melanotos	Charadriiformes	8.70	NA	1.02	79.73	Op	Mi	An	Te	D
Calidris_minutilla	Charadriiformes	6.13	NA	0.55	22.88	Op	Mi	An	Te	D
Calidris_pusilla	Charadriiformes	6.60	NA	0.51	27.50	Op	Mi	An	Te	D
Calidris_tenuirostris	Charadriiformes	9.80	NA	1.54	192.00	Op	Mi	An	Te	D
Callipepla_gambelii	Galliformes	9.30	11.60	1.32	165.95	So	Se	Pl	Te	D
Callipepla_squamata	Galliformes	10.00	11.15	1.45	183.86	Op	Se	Pl	Te	D
Callocephalon_fimbriatum	Psittaciformes	12.30	14.90	7.48	256.00	So	Pm	Pl	In	D
Caloenas_nicobarica	Columbiformes	14.20	17.20	3.06	537.86	De	Pm	Pl	Te	D
Calorhamphus_fuliginosus	Piciformes	9.05	10.60	1.10	42.60	De	Se	Pl	In	D
Calyptorhynchus_banksii	Psittaciformes	15.80	18.05	11.95	721.90	So	Se	Pl	In	D
Calyptorhynchus_funereus	Psittaciformes	16.60	18.90	15.08	674.72	So	Se	Pl	In	D
Campephaga_phoenicea	Passeriformes	9.50	12.30	1.05	27.10	So	Se	An	In	D
Campethera_nivosa	Piciformes	7.80	9.75	1.58	37.80	De	Se	An	In	D
Camptostoma_obsoletum	Passeriformes	5.40	6.75	0.36	8.10	So	Se	An	In	D
Campylopterus_largipennis	Caprimulgiformes	5.40	6.60	0.31	8.30	De	Se	Pl	Ae	D
Campylorhynchus_brunneicapillus	Passeriformes	9.00	10.63	1.34	38.90	Op	Se	An	In	D
Campylorhynchus_griseus	Passeriformes	8.80	10.20	1.31	42.40	So	Se	An	In	D
Caprimulgus_carolinensis	Caprimulgiformes	16.00	NA	1.36	109.00	So	Mi	An	Ae	N
Caprimulgus_europaeus	Caprimulgiformes	11.60	NA	0.88	67.00	So	Mi	An	Ae	N
Caprimulgus_macrurus	Caprimulgiformes	12.95	NA	1.01	66.20	So	Se	An	Ge	N
Caprimulgus_vociferus	Caprimulgiformes	12.80	NA	0.82	53.30	De	Mi	An	Ge	N
Cardellina_rubrifrons	Passeriformes	6.40	7.20	0.43	8.10	So	Mi	An	In	D
Cardinalis_cardinalis	Passeriformes	8.80	10.78	1.52	42.64	So	Se	Pl	In	D
Carduelis_carduelis	Passeriformes	5.60	6.70	0.65	16.00	So	Se	Pl	In	D
Carduelis_chloris	Passeriformes	6.80	8.00	0.86	26.00	So	Se	Pl	Ge	D
Carduelis_flammea	Passeriformes	5.00	6.05	0.59	13.00	De	Pm	Pl	Ge	D

Carduelis_tristis	Passeriformes	5.20	6.40	0.54	12.79	De	Se	Pl	In	D
Cariama_cristata	Cariamiformes	19.66	23.91	11.15	1400.00	So	Se	An	Te	D
Carpodacus_mexicanus	Passeriformes	5.70	7.65	0.78	21.40	So	Pm	Pl	In	D
Carpodacus_purpureus	Passeriformes	6.90	8.40	0.90	23.30	So	Pm	Pl	In	D
Casuarius_bennetti	Struthioniformes	28.20	34.90	23.65	34999.99	De	Se	Pl	Te	D
Casuarius_casuarius	Struthioniformes	32.80	41.55	36.35	44000.00	De	Se	Pl	Te	D
Cathartes_aura	Cathartiformes	18.65	NA	9.98	1518.24	Op	Pm	Ca/Re	Te	D
Catharus_fuscescens	Passeriformes	8.70	10.65	0.92	31.90	De	Mi	An	Te	D
Catharus_guttatus	Passeriformes	9.05	10.85	0.79	30.10	De	Mi	Om	Ge	D
Catharus_minimus	Passeriformes	8.80	10.63	0.77	31.58	So	Mi	An	Te	D
Catoptrophorus_semipalmatus	Charadriiformes	11.30	NA	2.33	245.70	Op	Mi	An	Te	D
Celeus_castaneus	Piciformes	9.40	11.45	2.11	85.59	De	Se	An	In	D
Centropus_phasianinus	Cuculiformes	14.40	18.05	4.12	381.98	So	Se	An	Te	D
Centropus_senegalensis	Cuculiformes	12.70	15.25	2.61	169.00	So	Se	An	Te	D
Centropus_sinensis	Cuculiformes	15.60	18.95	4.59	280.70	So	Se	An	Te	D
Ceratogymna_atrata	Bucerotiformes	19.00	21.90	13.50	1194.79	De	Se	Pl	In	D
Ceratogymna_elata	Bucerotiformes	21.80	25.70	14.29	1917.02	De	Se	Pl	In	D
Cercomacra_tyrannina	Passeriformes	7.80	9.05	0.74	16.30	De	Se	An	In	D
Certhia_familiaris	Passeriformes	5.23	6.65	0.39	9.00	De	Se	Om	In	D
Certhiaxis_cinnamomeus	Passeriformes	7.20	7.40	0.69	15.20	So	Se	An	In	D
Certhionyx_niger	Passeriformes	5.80	6.40	0.37	9.90	So	Mi	Om	In	D
Ceryle_rudis	Coraciiformes	11.30	14.80	1.59	84.37	Op	Se	An	In	D
Cettia_cetti	Passeriformes	6.60	7.90	0.58	13.22	So	Se	An	Ge	D
Ceuthmochares_aereus	Cuculiformes	12.15	14.25	1.33	65.80	De	Se	An	In	D
Ceyx_lepidus	Coraciiformes	7.45	9.18	0.56	21.32	De	Se	An	In	D
Ceyx_pictus	Coraciiformes	6.80	8.45	0.43	12.72	De	Se	An	In	D
Chaetura_pelagica	Caprimulgiformes	9.00	10.20	0.46	23.60	De	Mi	An	Ae	N
Chalcophaps_indica	Columbiformes	9.83	12.45	1.25	136.54	De	Se	Pl	Ge	D
Chalcopsitta_atra	Psittaciformes	10.50	12.90	5.40	195.00	Op	Se	Pl	In	D
Chamaea_fasciata	Passeriformes	7.20	8.55	0.77	14.73	So	Se	Om	In	D
Chamaeza_campanisona	Passeriformes	11.30	14.95	1.83	90.58	De	Se	An	Te	D
Charadrius_alexandrinus	Charadriiformes	10.00	NA	0.89	42.30	Op	Pm	An	Te	D

Charadrius_collaris	Charadriiformes	8.20	NA	0.77	28.30	Op	Pm	An	Te	D
Charadrius_dubius	Charadriiformes	8.50	NA	0.97	38.70	Op	Mi	An	Te	D
Charadrius_melodus	Charadriiformes	7.45	NA	0.99	54.39	Op	Mi	An	Te	D
Charadrius_modestus	Charadriiformes	11.10	NA	1.24	78.00	Op	Mi	An	Te	D
Charadrius_semipalmatus	Charadriiformes	9.10	NA	0.96	46.74	Op	Mi	An	Te	D
Charadrius_vociferus	Charadriiformes	10.60	NA	1.12	96.44	Op	Mi	An	Te	D
Charmosyna_papou	Psittaciformes	9.30	10.80	3.45	82.89	De	Se	Pl	In	D
Chelidoptera_tenebrosa	Piciformes	8.65	9.93	0.85	35.90	Op	Se	An	In	D
Cheramoeca_leucosterna	Passeriformes	7.70	8.25	0.41	14.10	Op	Se	An	Ae	D
Chersomanes_albofasciata	Passeriformes	8.20	9.65	0.94	24.61	Op	Se	An	Te	D
Chiroxiphia_caudata	Passeriformes	8.10	10.25	0.80	25.60	De	Se	Om	In	D
Chiroxiphia_lanceolata	Passeriformes	7.30	9.05	0.57	18.50	De	Se	Pl	In	D
Chiroxiphia_linearis	Passeriformes	7.80	9.15	0.68	17.44	De	Se	Pl	In	D
Chlamydera_maculata	Passeriformes	13.00	15.85	3.80	139.99	So	Se	Pl	In	D
Chlamydera_nuchalis	Passeriformes	13.60	15.35	5.01	200.43	So	Se	Pl	In	D
Chlamydotis_undulata	Otidiformes	21.30	27.70	5.31	1501.33	Op	Se	Pl	Te	D
Chloroceryle_amazona	Coraciiformes	12.80	15.40	1.95	126.38	Op	Se	An	In	D
Chloroceryle_americana	Coraciiformes	9.80	11.65	0.92	33.73	Op	Se	An	In	D
Chloropsis_hardwickii	Passeriformes	7.50	9.90	0.73	32.29	De	Mi	Om	In	D
Chlorospingus_ophthalmicus	Passeriformes	7.20	8.30	0.98	19.98	De	Se	An	In	D
Chlorothraupis_carmioli	Passeriformes	9.65	11.18	1.30	37.60	De	Se	An	In	D
Chordeiles_minor	Caprimulgiformes	12.30	NA	0.85	79.30	Op	Mi	An	Ae	N
Chrysococcyx_basalis	Cuculiformes	8.50	9.20	0.62	23.12	So	Se	An	Ge	D
Chrysococcyx_caprius	Cuculiformes	9.00	10.50	0.77	32.79	So	Se	An	In	D
Chrysococcyx_lucidus	Cuculiformes	8.30	10.30	0.69	24.44	So	Se	An	In	D
Chrysocolaptes_lucidus	Piciformes	10.97	13.77	4.75	142.00	So	Se	An	In	D
Chrysolophus_amherstiae	Galliformes	14.40	18.05	3.76	732.47	De	Se	Om	Te	D
Chthonicola_sagittatus	Passeriformes	6.90	8.20	0.60	13.29	So	Se	An	Te	D
Chunga_burmeisteri	Cariamiformes	19.90	23.40	7.24	1298.00	So	Se	An	Te	D
Cicinnurus_regius	Passeriformes	9.55	11.10	1.74	51.63	De	Se	Om	In	D
Ciconia_ciconia	Ciconiiformes	22.20	NA	14.67	3445.80	Op	Mi	An	Te	D
Ciconia_nigra	Ciconiiformes	19.80	NA	11.39	2926.00	So	Mi	An	Te	D

Cinclidium_leucurum	Passeriformes	8.40	11.15	0.89	26.83	So	Se	Om	Ge	D
Cinclodes_fuscus	Passeriformes	7.80	9.60	0.88	30.00	Op	Mi	An	Te	D
Cincloramphus_cruralis	Passeriformes	10.05	12.05	1.12	49.22	Op	Mi	An	Te	D
Cinclosoma_cinnamomeum	Passeriformes	9.10	11.65	1.47	56.14	So	Se	An	Te	D
Cinclus_cinclus	Passeriformes	7.50	9.60	1.32	61.47	Op	Se	An	Ge	D
Cinclus_mexicanus	Passeriformes	8.10	9.75	1.40	56.68	Op	Se	An	Ge	D
Cinnyricinclus_leucogaster	Passeriformes	8.90	10.25	1.18	44.00	So	Mi	Pl	In	D
Circaetus_gallicus	Accipitriformes	32.50	NA	13.00	1699.12	Op	Mi	An	Ae	D
Circus_aeruginosus	Accipitriformes	20.30	NA	5.48	704.07	Op	Pm	An	Ae	D
Circus_cyaneus	Accipitriformes	18.50	NA	4.78	392.98	Op	Mi	An	Ae	D
Cissa_chinensis	Passeriformes	14.60	16.40	3.45	126.64	De	Se	An	Ge	D
Cissopis_leverianus	Passeriformes	9.80	11.40	1.71	76.00	So	Se	Om	In	D
Cistothorus_palustris	Passeriformes	5.90	7.45	0.52	10.80	Op	Pm	An	In	D
Clamator_glandarius	Cuculiformes	12.50	14.50	1.90	143.00	So	Mi	An	In	D
Clangula_hyemalis	Anseriformes	13.50	NA	4.88	871.00	Op	Mi	An	Aq	D
Claravis_pretiosa	Columbiformes	7.80	10.55	0.90	68.20	De	Pm	Pl	Ge	D
Climacteris_erythrops	Passeriformes	6.40	8.05	0.88	23.30	So	Se	An	In	D
Climacteris_melanurus	Passeriformes	7.20	8.60	0.86	31.85	So	Se	An	In	D
Climacteris_picumnus	Passeriformes	7.65	9.75	1.00	28.94	So	Se	An	In	D
Climacteris_rufus	Passeriformes	8.40	9.80	1.03	32.64	So	Se	An	In	D
Clytorhynchus_pachycephaloides	Passeriformes	8.00	9.55	1.05	26.30	De	Se	An	In	D
Cnemophilus_loriae	Passeriformes	10.93	13.90	2.15	83.97	De	Se	Pl	In	D
Cnemotriccus_fuscatus	Passeriformes	7.05	8.53	0.46	13.60	So	Pm	An	In	D
Coccothraustes_coccothraustes	Passeriformes	8.50	10.00	1.42	56.63	De	Pm	Pl	In	D
Coccothraustes_vespertinus	Passeriformes	8.00	9.70	1.70	57.30	De	Pm	Pl	In	D
Coccyzus_americanus	Cuculiformes	9.33	11.49	1.17	64.00	De	Mi	An	In	D
Coccyzus_erythropthalmus	Cuculiformes	8.73	10.92	1.01	50.90	De	Mi	An	In	D
Coccyzus_minor	Cuculiformes	12.10	13.90	1.16	63.89	So	Pm	An	In	D
Coccyzus_vetula	Cuculiformes	12.20	14.90	2.00	96.00	De	Se	An	In	D
Coereba_flaveola	Passeriformes	4.90	6.43	0.44	10.01	So	Se	Pl	In	D
Colaptes_auratus	Piciformes	10.70	12.80	3.06	131.46	So	Pm	An	Ge	D
Colaptes_rubiginosus	Piciformes	8.80	10.60	2.19	55.79	So	Se	An	In	D

Colinus_virginianus	Galliformes	9.90	11.95	1.30	171.89	So	Se	Pl	Te	D
Colius_striatus	Coliiformes	7.60	9.46	1.14	51.10	So	Se	Pl	In	D
Collocalia_esculenta	Caprimulgiformes	4.98	5.90	0.20	6.29	De	Se	An	Ae	D
Collocalia_spodiopygia	Caprimulgiformes	7.60	8.25	0.24	8.03	De	Se	An	Ae	D
Colluricincla_harmonica	Passeriformes	11.00	12.90	2.00	65.80	So	Se	An	In	D
Colluricincla_megarhyncha	Passeriformes	8.90	10.95	1.34	32.34	De	Se	An	In	D
Colonia_colonus	Passeriformes	7.80	8.90	0.47	18.30	De	Se	An	In	D
Columba_livia	Columbiformes	11.13	13.90	2.04	354.20	Op	Se	Pl	Te	D
Columba_palumbus	Columbiformes	12.40	NA	2.51	490.00	So	Se	Pl	Te	D
Columbina_inca	Columbiformes	6.90	9.35	0.73	47.50	Op	Se	Pl	Te	D
Columbina_minuta	Columbiformes	7.45	9.00	0.56	32.59	So	Se	Pl	Te	D
Columbina_picui	Columbiformes	7.80	9.70	0.75	47.00	Op	Mi	Pl	Te	D
Columbina_talpacoti	Columbiformes	7.25	9.60	0.72	46.04	Op	Pm	Pl	Te	D
Conopophaga_lineata	Passeriformes	9.33	11.05	0.70	25.21	De	Se	An	In	D
Conopophila_albogularis	Passeriformes	5.60	6.65	0.55	11.80	So	Se	Om	In	D
Conopophila_rufogularis	Passeriformes	5.60	6.55	0.44	10.80	So	Se	Om	In	D
Contopus_cooperi	Passeriformes	9.20	11.00	0.64	32.10	De	Mi	An	In	D
Contopus_latirostris	Passeriformes	7.10	8.20	0.38	11.20	De	Se	An	In	D
Contopus_sordidulus	Passeriformes	7.80	8.60	0.34	13.10	De	Mi	An	In	D
Contopus_virens	Passeriformes	7.60	8.90	0.45	13.90	De	Mi	An	In	D
Copsychus_malabaricus	Passeriformes	9.50	11.10	0.87	29.23	De	Se	An	In	D
Copsychus_saularis	Passeriformes	9.20	11.10	1.10	36.00	De	Se	An	Te	D
Coracias_caudatus	Coraciiformes	13.80	15.70	2.06	110.00	So	Pm	An	In	D
Coracias_garrulus	Coraciiformes	12.95	16.10	2.32	146.00	So	Mi	An	In	D
Coracina_maxima	Passeriformes	13.30	15.75	2.20	127.00	Op	Se	An	Te	D
Coracina_novaehollandiae	Passeriformes	15.70	18.45	2.40	118.00	So	Se	An	In	D
Coracina_papuensis	Passeriformes	12.60	15.00	2.03	63.45	So	Se	An	In	D
Coracopsis_vasa	Psittaciformes	14.60	17.50	8.68	525.00	De	Se	Pl	In	D
Coragyps_atratus	Cathartiformes	16.80	NA	11.99	1881.69	Op	Pm	Om	Te	D
Corcorax_melanorhamphos	Passeriformes	13.77	15.87	5.20	364.00	So	Se	Om	Te	D
Cormobates_leucophaea	Passeriformes	7.00	8.00	0.81	22.00	So	Se	An	In	D
Corvus_albus	Passeriformes	16.30	19.55	8.75	524.00	Op	Pm	An	Te	D

Corvus_bennetti	Passeriformes	15.70	18.75	6.44	389.00	So	Se	Om	Ge	D
Corvus_brachyrhynchos	Passeriformes	13.81	15.05	7.17	448.76	So	Pm	Om	Te	D
Corvus_corax	Passeriformes	19.45	23.20	14.45	927.97	Op	Pm	Om	Te	D
Corvus_corone	Passeriformes	15.63	18.70	8.51	570.00	Op	Se	An	Te	D
Corvus_coronoides	Passeriformes	17.40	20.60	9.83	644.30	So	Se	Om	Ge	D
Corvus_cryptoleucus	Passeriformes	17.80	20.35	8.95	533.54	Op	Se	An	Te	D
Corvus_frugilegus	Passeriformes	15.00	17.85	7.61	452.10	Op	Se	Om	Te	D
Corvus_macrorhynchos	Passeriformes	15.85	19.03	12.66	513.14	So	Se	Om	Ge	D
Corvus_mellori	Passeriformes	17.20	20.10	8.50	540.37	So	Se	Om	Ge	D
Corvus_monedula	Passeriformes	12.60	14.70	5.15	246.00	Op	Pm	Om	Te	D
Corvus_moneduloides	Passeriformes	13.10	16.50	7.23	277.38	So	Se	Om	Ge	D
Corvus_orru	Passeriformes	16.40	19.50	8.91	507.96	So	Se	Om	Ge	D
Corvus_ruficollis	Passeriformes	18.30	21.40	10.14	573.50	Op	Se	Om	Te	D
Corvus_splendens	Passeriformes	14.50	16.35	5.91	292.55	Op	Se	Om	Te	D
Corydon_sumatranus	Passeriformes	14.00	16.60	2.40	140.00	De	Se	An	In	D
Coryphistera_alaudina	Passeriformes	8.30	9.80	0.94	30.00	So	Se	An	Te	D
Coryphospingus_cucullatus	Passeriformes	6.30	7.63	0.67	14.28	De	Se	Pl	Ge	D
Corythaeola_cristata	Musophagiformes	15.40	20.10	5.98	964.99	De	Se	Pl	In	D
Corythaixoides_concolor	Musophagiformes	13.30	16.15	3.78	258.00	So	Se	Pl	In	D
Coturnix_chinensis	Galliformes	5.55	7.45	0.56	40.41	So	Se	Pl	Te	D
Coturnix_coturnix	Galliformes	8.30	10.05	0.94	96.28	Op	Mi	Pl	Te	D
Coua_caerulea	Cuculiformes	15.80	18.00	4.05	244.00	De	Se	An	In	D
Cracticus_nigrogularis	Passeriformes	13.15	16.35	3.02	128.00	So	Se	An	In	D
Cracticus_quoyi	Passeriformes	13.80	16.60	3.55	168.00	De	Se	An	In	D
Cracticus_torquatus	Passeriformes	12.85	15.18	2.78	82.88	So	Se	Om	In	D
Crax_alector	Galliformes	20.20	24.73	6.76	2872.00	De	Se	Pl	Te	D
Crax_rubra	Galliformes	20.30	26.00	9.56	4133.00	De	Se	Pl	Te	D
Crinifer_piscator	Musophagiformes	15.30	18.45	4.70	370.00	Op	Se	Pl	In	D
Crossoptilon_auritum	Galliformes	16.20	20.20	5.69	1768.33	De	Se	Pl	Te	D
Crotophaga_ani	Cuculiformes	10.35	13.20	1.56	110.09	Op	Se	An	In	D
Crypturellus_soui	Struthioniformes	11.40	13.70	1.49	216.16	De	Se	Pl	Te	D
Crypturellus_tataupa	Struthioniformes	9.45	12.25	1.26	218.80	So	Se	Pl	Te	D

Cuculus_canorus	Cuculiformes	11.50	15.25	1.57	111.36	So	Mi	An	In	D
Cuculus_pallidus	Cuculiformes	11.70	15.15	1.43	87.69	So	Se	An	In	D
Cuculus_saturatus	Cuculiformes	11.80	14.80	1.58	87.00	De	Mi	An	In	D
Cursorius_cursor	Charadriiformes	12.80	16.30	1.44	138.00	Op	Pm	An	Te	D
Cursorius_temminckii	Charadriiformes	10.30	13.20	1.25	69.20	Op	Se	An	Te	D
Cyanerpes_cyaneus	Passeriformes	6.10	7.10	0.55	14.00	De	Se	Om	In	D
Cyanocitta_cristata	Passeriformes	11.63	13.93	2.92	88.00	So	Se	Om	Te	D
Cyanocitta_stelleri	Passeriformes	12.30	14.10	3.54	128.00	De	Se	Om	In	D
Cyanocorax_affinis	Passeriformes	12.80	16.25	4.36	212.00	De	Se	Om	In	D
Cyanocorax_chrysops	Passeriformes	9.35	10.60	4.15	166.00	De	Se	An	In	D
Cyanocorax_morio	Passeriformes	14.50	16.80	4.80	204.00	So	Se	Om	In	D
Cyanocorax_yncas	Passeriformes	11.50	13.70	2.32	78.50	So	Se	Om	In	D
Cyanoliseus_patagonus	Psittaciformes	11.40	13.10	8.23	277.88	So	Pm	Pl	In	D
Cyanopica_cyanus	Passeriformes	11.00	13.05	2.80	95.91	So	Se	Om	Ge	D
Cyanoramphus_unicolor	Psittaciformes	9.30	11.10	4.03	149.03	So	Se	Pl	Te	D
Cyclarhis_gujanensis	Passeriformes	8.78	10.39	1.26	28.80	So	Se	An	In	D
Cyclopsitta_diophthalma	Psittaciformes	8.20	9.35	1.70	41.84	De	Se	Pl	In	D
Cygnus_cygnus	Anseriformes	18.45	NA	19.50	9349.99	Op	Mi	Pl	Aq	D
Cygnus_olor	Anseriformes	16.90	NA	16.64	10682.04	Op	Mi	Pl	Aq	D
Cymbilaimus_lineatus	Passeriformes	9.60	11.80	1.22	35.80	De	Se	An	In	D
Cyornis_rubeculoides	Passeriformes	8.00	9.55	0.55	14.20	So	Se	An	In	D
Cyphorhinus_arada	Passeriformes	7.30	8.70	0.91	20.90	De	Se	An	Ge	D
Cypsiurus_parvus	Caprimulgiformes	7.93	9.03	0.30	13.60	De	Se	An	Ae	D
Cyrtonyx_montezumae	Galliformes	9.80	12.40	1.46	185.38	So	Se	Pl	Te	D
Dacelo_gaudichaud	Coraciiformes	15.80	18.60	2.15	143.00	De	Se	An	In	D
Dacelo_leachii	Coraciiformes	20.30	22.55	4.23	307.98	So	Se	An	In	D
Dacelo_novaeguineae	Coraciiformes	19.80	23.05	4.31	333.80	So	Se	An	In	D
Dacnis_cayana	Passeriformes	6.30	7.55	0.55	13.00	So	Se	Pl	In	D
Daphoenositta_chrysoptera	Passeriformes	5.93	6.97	0.61	11.70	So	Se	An	In	D
Daptrius_ater	Falconiformes	17.80	NA	5.28	351.75	So	Pm	An	Te	D
Dasyornis_broadbenti	Passeriformes	9.90	11.98	1.88	67.90	So	Se	Om	Ge	D
Delichon_urbicum	Passeriformes	7.80	8.55	0.48	14.50	Op	Mi	An	Ae	D

Dendragapus_canadensis	Galliformes	13.30	16.15	2.44	473.65	De	Se	Pl	Te	D
Dendrocincla_fuliginosa	Passeriformes	8.95	10.63	1.06	38.70	De	Se	An	In	D
Dendrocitta_formosae	Passeriformes	11.70	14.20	2.49	102.74	So	Se	Om	In	D
Dendrocitta_vagabunda	Passeriformes	12.50	14.60	2.70	100.00	So	Se	An	Te	D
Dendrocolaptes_certhia	Passeriformes	11.65	13.93	1.74	68.70	De	Se	An	In	D
Dendrocolaptes_platyrostris	Passeriformes	11.20	13.00	1.32	61.70	De	Se	An	In	D
Dendrocopos_maculatus	Piciformes	6.40	8.45	1.02	24.50	De	Se	An	In	D
Dendrocopos_major	Piciformes	9.80	NA	2.70	74.94	De	Se	Om	In	D
Dendrocopos_minor	Piciformes	7.00	8.90	1.16	19.80	De	Se	An	In	D
Dendroica_caerulescens	Passeriformes	6.00	7.65	0.43	10.14	De	Mi	An	In	D
Dendroica_coronata	Passeriformes	6.40	7.70	0.51	11.94	De	Mi	An	In	D
Dendroica_magnolia	Passeriformes	6.20	7.05	0.37	8.14	De	Mi	An	In	D
Dendroica_petechia	Passeriformes	6.70	7.80	0.52	10.22	De	Mi	An	In	D
Dendropicos_fuscescens	Piciformes	6.70	8.10	1.22	26.00	So	Se	An	In	D
Dendroplex_picus	Passeriformes	6.80	8.65	1.30	41.34	So	Se	An	In	D
Deroptyus_accipitrinus	Psittaciformes	13.20	16.45	7.74	246.00	De	Se	Pl	In	D
Dicrurus_hottentottus	Passeriformes	11.30	14.25	1.96	79.19	De	Se	An	In	D
Dicrurus_remifer	Passeriformes	10.60	12.20	1.16	43.10	De	Se	An	In	D
Didunculus_strigirostris	Columbiformes	12.50	15.25	2.69	400.00	De	Se	Pl	In	D
Dinopium_benghalense	Piciformes	9.80	12.05	2.80	100.00	So	Se	An	In	D
Dolichonyx_oryzivorus	Passeriformes	7.00	8.42	1.07	31.46	De	Mi	Om	Ge	D
Donacobius_atricapilla	Passeriformes	9.40	10.70	1.15	36.80	Op	Se	An	In	D
Dromaius_novaehollandiae	Struthioniformes	25.40	30.10	28.88	34093.25	Op	Se	Pl	Te	D
Dromas_ardeola	Charadriiformes	16.60	NA	3.76	325.00	Op	Se	An	Te	D
Drymodes_brunneopygia	Passeriformes	8.20	10.60	0.95	32.95	So	Se	An	Ge	D
Dryocopus_javensis	Piciformes	11.80	15.78	6.07	271.40	De	Se	An	In	D
Dryocopus_martius	Piciformes	10.70	13.00	7.43	321.00	De	Se	An	In	D
Dryocopus_pileatus	Piciformes	12.80	16.25	6.80	286.59	De	Se	An	In	D
Dryoscopus_cubla	Passeriformes	8.70	10.25	1.13	26.23	So	Se	An	In	D
Ducula_aenea	Columbiformes	13.40	16.50	3.06	545.00	De	Se	Pl	In	D
Ducula_badia	Columbiformes	13.90	17.30	3.05	485.99	So	Se	Pl	In	D
Dulus_dominicus	Passeriformes	8.07	9.98	1.27	48.40	So	Se	Pl	In	D

Dumetella_carolinensis	Passeriformes	7.68	9.20	1.14	35.30	De	Mi	Om	In	D
Dysithamnus_mentalis	Passeriformes	8.05	9.38	0.70	14.87	De	Se	An	In	D
Eclectus_roratus	Psittaciformes	13.80	16.60	7.36	480.76	De	Se	Pl	In	D
Elaenia_albiceps	Passeriformes	6.78	8.33	0.51	15.50	So	Pm	Om	In	D
Elaenia_frantzii	Passeriformes	6.80	8.00	0.61	19.60	Op	Se	Pl	In	D
Elaenia_martinica	Passeriformes	8.20	9.38	0.65	20.79	So	Se	Om	In	D
Elanus_caeruleus	Accipitriformes	18.10	NA	3.43	259.76	Op	Pm	An	Ae	D
Elanus_leucurus	Accipitriformes	21.05	NA	2.96	346.00	Op	Pm	An	Ae	D
Empidonax_minimus	Passeriformes	7.03	8.47	0.37	10.00	De	Mi	An	In	D
Empidonax_oberholseri	Passeriformes	7.47	8.57	0.41	10.40	So	Mi	An	In	D
Empidonax_virescens	Passeriformes	7.43	7.63	0.43	12.60	So	Mi	An	In	D
Enicognathus_ferrugineus	Psittaciformes	10.40	13.10	5.32	160.00	So	Se	Pl	In	D
Enicurus_scouleri	Passeriformes	6.80	7.95	0.76	17.80	Op	Se	An	Te	D
Entomyzon_cyanotis	Passeriformes	10.70	12.55	2.23	105.26	So	Se	Om	In	D
Eopsaltria_australis	Passeriformes	8.00	10.85	0.86	20.15	So	Se	An	In	D
Eopsaltria_griseogularis	Passeriformes	9.10	10.95	0.83	19.38	So	Se	An	In	D
Eopsaltria_pulverulenta	Passeriformes	7.80	9.35	0.83	19.19	So	Pm	An	In	D
Eos_bornea	Psittaciformes	10.40	12.20	4.78	156.00	De	Se	Pl	In	D
Eos_cyanogenia	Psittaciformes	10.00	12.40	4.85	167.00	De	Se	Pl	In	D
Epinecrophylla_fulviventris	Passeriformes	5.70	7.00	0.48	10.40	De	Se	An	In	D
Epthianura_albifrons	Passeriformes	5.70	7.45	0.53	13.30	Op	Se	An	Ge	D
Epthianura_aurifrons	Passeriformes	6.00	7.30	0.43	10.50	So	Se	An	Ge	D
Epthianura_tricolor	Passeriformes	6.00	7.15	0.44	10.70	So	Se	An	Ge	D
Eremophila_alpestris	Passeriformes	7.38	8.44	0.89	33.33	Op	Mi	Om	Te	D
Eremophila_bilopha	Passeriformes	7.30	8.80	0.82	38.50	Op	Pm	Pl	Te	D
Erithacus_rubecula	Passeriformes	7.30	9.15	0.64	17.70	De	Mi	An	Ge	D
Erythrura_gouldiae	Passeriformes	5.20	6.40	0.53	13.80	Op	Se	Pl	Ge	D
Erythrura_trichroa	Passeriformes	5.20	6.95	0.58	14.39	So	Se	Pl	In	D
Esacus_giganteus	Charadriiformes	22.70	27.45	5.35	1015.87	Op	Pm	An	Te	D
Estrilda_astrild	Passeriformes	4.80	5.40	0.34	8.29	Op	Se	Pl	Ge	D
Estrilda_caerulescens	Passeriformes	5.20	6.05	0.41	9.40	So	Se	Pl	Ge	D
Estrilda_melpoda	Passeriformes	5.00	5.60	0.38	7.60	Op	Se	Om	Ge	D

Eubucco_bourcierii	Piciformes	7.90	9.30	1.03	37.00	De	Se	Pl	In	D
Eudromia_elegans	Struthioniformes	11.80	17.00	2.49	702.90	So	Se	Pl	Te	D
Eudynamys_scolopaceus	Cuculiformes	12.50	14.45	2.46	194.92	So	Pm	Pl	In	D
Eudynamys_taitensis	Cuculiformes	12.00	14.30	2.00	117.00	De	Mi	An	In	D
Eudyptes_chrysocome	Sphenisciformes	22.70	NA	12.42	2327.85	Op	Pm	An	Aq	D
Eulampis_holosericeus	Caprimulgiformes	4.50	5.55	0.24	5.70	So	Se	Pl	Ae	D
Eulampis_jugularis	Caprimulgiformes	5.10	6.35	0.32	8.85	De	Se	Pl	Ae	D
Euneornis_campestris	Passeriformes	3.10	7.60	0.70	16.00	So	Se	Pl	In	D
Eunymphicus_cornutus	Psittaciformes	11.50	12.95	3.33	130.00	De	Se	Pl	In	D
Euphagus_carolinus	Passeriformes	8.30	10.00	1.51	59.57	So	Mi	Om	Te	D
Euplectes_orix	Passeriformes	6.40	7.50	0.76	22.97	Op	Se	Om	Te	D
Eupodotis_afra	Otidiformes	14.70	17.38	3.76	689.99	Op	Se	Om	Te	D
Eurocephalus_anguitimens	Passeriformes	10.30	11.93	1.73	69.04	So	Se	An	In	D
Eurostopodus_macrotis	Caprimulgiformes	17.45	NA	1.46	140.64	De	Se	An	Ae	N
Eurypyga_helias	Eurypygiformes	12.87	15.92	2.48	210.00	So	Se	An	Te	D
Eurystomus_glaucurus	Coraciiformes	14.15	17.00	1.74	110.00	So	Se	An	Ge	D
Eurystomus_orientalis	Coraciiformes	14.80	17.63	1.98	143.02	So	Mi	An	Ae	D
Falco_berigora	Falconiformes	20.40	NA	6.24	587.01	Op	Se	An	Ge	D
Falco_biarmicus	Falconiformes	18.80	NA	6.08	606.09	Op	Pm	An	Ae	D
Falco_cenchroides	Falconiformes	15.30	NA	3.14	170.47	Op	Pm	An	Ge	D
Falco_eleonorae	Falconiformes	16.40	NA	4.04	390.00	Op	Mi	An	Ae	D
Falco_naumanni	Falconiformes	11.90	NA	2.71	152.06	Op	Mi	An	Ge	D
Falco_novaeseelandiae	Falconiformes	17.80	NA	5.50	418.60	Op	Pm	An	Ae	D
Falco_peregrinus	Falconiformes	20.50	NA	6.19	759.95	Op	Pm	An	Ae	D
Falco_rufigularis	Falconiformes	15.70	NA	3.18	163.64	So	Se	An	Ae	D
Falco_rusticolus	Falconiformes	22.90	NA	9.43	1431.72	Op	Mi	An	Ae	D
Falco_sparverius	Falconiformes	11.95	NA	2.49	114.61	Op	Mi	An	In	D
Falco_subbuteo	Falconiformes	14.70	NA	3.59	208.17	Op	Mi	An	Ae	D
Falco_tinnunculus	Falconiformes	16.60	NA	3.87	183.21	Op	Mi	An	Ae	D
Falco_vespertinus	Falconiformes	13.50	NA	2.70	151.49	Op	Mi	An	Ae	D
Falcunculus_frontatus	Passeriformes	7.50	9.28	1.24	27.94	De	Se	An	In	D
Ficedula_westermanni	Passeriformes	6.50	8.25	0.33	7.80	De	Se	An	In	D

Florisuga_mellivora	Caprimulgiformes	4.70	5.90	0.26	7.40	De	Se	Pl	Ae	D
Formicarius_analis	Passeriformes	10.30	12.55	1.30	62.19	De	Se	An	Te	D
Formicarius_colma	Passeriformes	9.40	11.45	1.08	47.00	De	Se	An	Te	D
Formicivora_grisea	Passeriformes	6.60	7.95	0.45	10.36	So	Se	An	In	D
Forpus_passerinus	Psittaciformes	6.30	7.40	1.10	23.00	So	Se	Pl	Ge	D
Foudia_madagascariensis	Passeriformes	6.20	7.40	0.75	16.59	Op	Se	Pl	In	D
Foulehaio_carunculatus	Passeriformes	7.40	8.85	0.98	37.00	De	Se	Pl	In	D
Francolinus_leucoscepus	Galliformes	13.00	16.30	3.20	640.61	So	Se	Pl	Te	D
Francolinus_sephaena	Galliformes	10.60	13.75	2.25	340.47	So	Se	Om	Te	D
Fregata_aquila	Suliformes	19.66	NA	9.12	1620.00	Op	Mi	An	Ae	D
Fringilla_coelebs	Passeriformes	6.75	8.13	0.81	23.81	So	Pm	Om	Ge	D
Fringilla_montifringilla	Passeriformes	6.20	8.13	0.75	23.19	De	Mi	Pl	Ge	D
Fulica_atra	Gruiformes	11.60	NA	2.85	717.12	Op	Se	Pl	Ge	D
Fulmarus_glacialis	Procellariiformes	16.10	NA	6.33	611.94	Op	Mi	An	Aq	D
Furnarius_rufus	Passeriformes	8.12	9.61	1.33	46.42	Op	Se	An	Te	D
Galbula_albirostris	Piciformes	8.50	10.85	0.61	22.10	De	Se	An	In	D
Galbula_galbula	Piciformes	8.80	10.05	0.58	23.00	So	Se	An	In	D
Galerida_cristata	Passeriformes	7.30	9.18	1.17	42.68	Op	Se	Om	Te	D
Gallicolumba_rubescens	Columbiformes	8.20	10.95	1.03	60.00	De	Se	Pl	In	D
Gallinago_gallinago	Charadriiformes	9.30	NA	1.41	112.94	Op	Mi	An	Te	D
Gallinago_nobilis	Charadriiformes	10.20	NA	2.09	192.44	Op	Se	An	Te	D
Gallinago_stenura	Charadriiformes	10.00	NA	1.50	113.00	Op	Mi	An	Te	D
Gallus_gallus	Galliformes	8.55	10.95	3.53	751.72	De	Se	Pl	Te	D
Garrulax_leucolophus	Passeriformes	11.10	12.95	2.46	123.44	So	Se	An	Te	D
Garrulus_glandarius	Passeriformes	14.05	16.53	3.91	159.46	De	Pm	Om	In	D
Garrulus_lanceolatus	Passeriformes	11.80	14.35	2.54	97.70	De	Pm	Om	In	D
Gavia_stellata	Gaviiformes	16.10	NA	5.54	1486.00	Op	Mi	An	Aq	D
Geococcyx_californianus	Cuculiformes	15.90	18.85	3.49	376.00	So	Se	An	Te	D
Geoffroyus_geoffroyi	Psittaciformes	11.90	14.50	4.26	164.00	De	Se	Pl	In	D
Geopelia_cuneata	Columbiformes	6.10	8.75	0.59	31.60	So	Se	Pl	Te	D
Geopelia_humeralis	Columbiformes	9.30	12.00	1.26	128.42	So	Se	Pl	Te	D
Geophaps_smithii	Columbiformes	8.30	12.45	1.39	194.00	De	Pm	Pl	Te	D

Geositta_cunicularia	Passeriformes	8.40	9.95	0.97	28.50	Op	Se	An	Te	D
Geothlypis_trichas	Passeriformes	5.60	7.45	0.52	9.54	De	Mi	An	In	D
Geotrygon_montana	Columbiformes	10.00	12.95	1.14	133.86	De	Se	Pl	Te	D
Gerygone_flavolateralis	Passeriformes	5.80	7.00	0.34	6.30	De	Se	An	In	D
Gerygone_fusca	Passeriformes	5.10	6.15	0.34	5.40	So	Se	An	In	D
Glareola_nuchalis	Charadriiformes	8.80	12.10	0.85	62.70	Op	Pm	An	Ae	D
Glaucidium_brasilianum	Strigiformes	12.30	NA	2.51	63.30	So	Se	An	In	D
Glaucidium_gnoma	Strigiformes	11.30	NA	3.60	61.25	So	Se	An	In	D
Glaucidium_passerinum	Strigiformes	12.60	NA	2.59	57.87	De	Pm	An	In	D
Glaucis_hirsutus	Caprimulgiformes	4.75	5.93	0.25	6.76	De	Se	Pl	Ae	D
Glossopsitta_concinna	Psittaciformes	8.10	10.05	2.87	70.54	So	Se	Pl	In	D
Glossopsitta_porphyrocephala	Psittaciformes	6.80	8.05	1.89	44.70	So	Se	Pl	In	D
Glossopsitta_pusilla	Psittaciformes	6.60	8.00	1.59	39.40	So	Se	Pl	In	D
Glyphorynchus_spirurus	Passeriformes	5.90	7.63	0.62	14.60	De	Se	An	In	D
Gnorimopsar_chopi	Passeriformes	8.40	9.28	1.80	65.90	So	Se	Om	Te	D
Goura_cristata	Columbiformes	18.00	22.70	5.27	2000.00	So	Se	Pl	Te	D
Goura_scheepmakeri	Columbiformes	18.00	21.10	5.75	2235.00	So	Se	Pl	Te	D
Goura_victoria	Columbiformes	17.40	22.60	5.43	2384.00	So	Se	Pl	Te	D
Gracula_religiosa	Passeriformes	11.70	14.20	3.68	192.00	De	Se	Pl	In	D
Grallina_bruijni	Passeriformes	8.40	9.70	1.00	38.40	So	Pm	An	Te	D
Grallina_cyanoleuca	Passeriformes	10.56	12.43	1.68	82.57	So	Se	An	Te	D
Graydidascalus_brachyurus	Psittaciformes	11.60	12.90	5.30	159.00	De	Se	Pl	In	D
Grus_canadensis	Gruiformes	18.58	NA	14.82	4296.22	Op	Mi	Om	Te	D
Grus_grus	Gruiformes	21.40	NA	19.96	5499.99	Op	Mi	Pl	Te	D
Guira_guira	Cuculiformes	10.93	13.05	1.92	141.00	Op	Se	An	Te	D
Guttera_pucherani	Galliformes	15.60	18.95	4.90	1149.00	De	Se	Om	Te	D
Gymnobucco_calvus	Piciformes	8.35	9.68	1.25	54.51	De	Pm	Pl	In	D
Gymnocichla_nudiceps	Passeriformes	8.50	10.60	1.06	30.50	De	Se	An	In	D
Gymnoderus_foetidus	Passeriformes	15.60	17.55	3.53	275.49	So	Se	Pl	In	D
Gymnogyps_californianus	Cathartiformes	25.50	NA	27.25	8442.74	Op	Se	Ca/Re	Te	D
Gymnophaps_albertisii	Columbiformes	12.50	15.85	2.10	259.00	De	Se	Pl	In	D
Gymnopithys_leucaspis	Passeriformes	8.50	9.70	0.76	27.20	De	Se	An	In	D

Gymnorhina_tibicen	Passeriformes	15.85	18.60	4.65	284.87	So	Se	An	Te	D
Gymnorhinus_cyanocephalus	Passeriformes	10.20	13.10	3.51	104.82	So	Pm	Om	Ge	D
Gypaetus_barbatus	Accipitriformes	30.95	NA	22.25	5694.98	Op	Pm	Ca/Re	Te	D
Gypohierax_angolensis	Accipitriformes	21.70	NA	10.13	1600.00	So	Se	Pl	In	D
Gyps_africanus	Accipitriformes	20.10	NA	18.25	5432.99	So	Se	Ca/Re	Te	D
Haematopus_ater	Charadriiformes	13.60	NA	5.15	724.00	Op	Se	An	Te	D
Haematopus_leucopodus	Charadriiformes	13.00	NA	4.55	617.99	Op	Se	An	Te	D
Haematopus_ostralegus	Charadriiformes	14.10	NA	4.52	526.00	Op	Se	An	Te	D
Halcyon_leucocephala	Coraciiformes	10.30	13.05	1.08	41.80	So	Pm	An	In	D
Halcyon_senegalensis	Coraciiformes	10.90	13.25	1.45	73.44	So	Se	An	In	D
Halcyon_smyrnensis	Coraciiformes	11.80	14.53	1.84	91.40	So	Pm	An	In	D
Haliaeetus_albicilla	Accipitriformes	30.20	NA	16.50	4729.27	Op	Pm	An	Ae	D
Haliaeetus_leucocephalus	Accipitriformes	28.15	NA	18.04	4700.58	Op	Mi	An	Ge	D
Haliaeetus_vocifer	Accipitriformes	21.60	NA	12.19	3400.00	So	Se	An	In	D
Harpactes_ardens	Trogoniformes	13.30	15.95	1.77	96.29	De	Se	An	In	D
Harpactes_erythrocephalus	Trogoniformes	12.87	15.00	1.92	80.29	De	Se	An	In	D
Harpagus_bidentatus	Accipitriformes	18.10	NA	3.83	215.25	So	Se	An	In	D
Heliothryx_barroti	Caprimulgiformes	4.60	5.70	0.23	5.50	De	Se	Pl	Ae	D
Helmitheros_vermivorum	Passeriformes	5.80	6.85	0.59	14.16	De	Mi	An	In	D
Hemiphaga_novaeseelandiae	Columbiformes	13.50	16.25	2.97	653.00	So	Se	Pl	In	D
Hemiprocne_comata	Caprimulgiformes	9.30	10.18	0.42	20.63	De	Se	An	In	D
Hemiprocne_mystacea	Caprimulgiformes	11.37	15.35	1.10	70.10	So	Se	An	Ae	D
Hemitriccus_margaritaceiventer	Passeriformes	6.58	7.70	0.45	8.40	So	Se	An	In	D
Henicophaps_albifrons	Columbiformes	11.40	14.65	2.10	247.00	Op	Se	Om	Te	D
Henicorhina_leucosticta	Passeriformes	6.40	8.05	0.78	14.18	De	Se	An	Ge	D
Herpetotheres_cachinnans	Falconiformes	21.25	NA	6.55	623.58	So	Se	An	In	D
Heteromyias_albispecularis	Passeriformes	10.00	12.80	1.05	36.30	De	Se	An	In	D
Heteroscelus_incanus	Charadriiformes	10.10	NA	1.29	108.24	Op	Mi	An	Te	D
Hieraaetus_morphnoides	Accipitriformes	23.40	NA	7.81	775.05	So	Se	An	Ge	D
Hieraaetus_pennatus	Accipitriformes	22.60	NA	5.31	823.75	De	Mi	An	Ge	D
Himantopus_himantopus	Charadriiformes	11.75	NA	1.90	176.82	Op	Pm	An	Te	D
Himatione_sanguinea	Passeriformes	5.80	6.77	0.67	15.17	De	Se	Pl	In	D

Hirundapus_caudacutus	Caprimulgiformes	12.80	13.85	1.29	95.50	De	Mi	An	Ae	D
Hirundo_ariel	Passeriformes	7.30	7.95	0.36	10.84	Op	Pm	An	Ae	D
Hirundo_nigricans	Passeriformes	7.60	8.70	0.52	15.40	So	Pm	An	Ae	D
Hirundo_rustica	Passeriformes	8.00	9.35	0.55	17.91	Op	Mi	An	Ae	D
Hirundo_senegalensis	Passeriformes	9.00	10.40	0.51	45.35	Op	Se	An	Ae	D
Hydrophasianus_chirurgus	Charadriiformes	9.40	NA	1.40	160.03	Op	Pm	Om	Te	D
Hylacola_cauta	Passeriformes	7.00	9.10	0.81	15.21	So	Se	An	Te	D
Hylocichla_mustelina	Passeriformes	8.26	9.84	1.25	50.09	De	Mi	Om	Ge	D
Hylophylax_naevius	Passeriformes	7.65	9.18	0.59	14.20	De	Se	An	In	D
Hymenops_perspicillatus	Passeriformes	8.40	9.85	0.76	22.90	Op	Mi	An	Te	D
Hypocnemis_cantator	Passeriformes	6.55	8.28	0.63	12.29	De	Se	An	In	D
Hypothymis_azurea	Passeriformes	7.00	8.65	0.52	11.10	De	Se	An	In	D
Ibidorhyncha_struthersii	Charadriiformes	11.65	NA	2.30	294.00	Op	Pm	An	Te	D
Icteria_virens	Passeriformes	8.30	9.95	0.91	24.89	So	Se	An	In	D
Icterus_cayanensis	Passeriformes	7.40	8.60	1.29	35.44	So	Se	An	In	D
Icterus_galbula	Passeriformes	8.40	9.70	1.13	32.83	De	Mi	Om	In	D
Icterus_spurius	Passeriformes	7.10	8.80	0.86	19.44	So	Mi	An	In	D
Ifrita_kowaldi	Passeriformes	8.60	10.00	1.25	32.10	De	Se	An	In	D
Illadopsis_fulvescens	Passeriformes	8.90	10.45	1.19	27.90	De	Se	An	In	D
Indicator_minor	Piciformes	5.80	7.75	0.67	28.20	So	Se	An	In	D
Irena_puella	Passeriformes	9.55	11.58	1.36	64.90	De	Se	Pl	In	D
Ithaginis_cruentus	Galliformes	12.30	14.25	3.18	530.67	So	Se	Pl	Te	D
Ixos_philippinus	Passeriformes	9.00	10.50	1.12	38.60	De	Se	Pl	In	D
Jacamerops_aureus	Piciformes	11.70	13.60	1.11	62.90	De	Se	An	In	D
Jacana_jacana	Charadriiformes	7.12	NA	1.29	106.24	Op	Se	Om	Te	D
Jacana_spinosa	Charadriiformes	8.90	NA	1.38	94.00	Op	Se	An	Te	D
Junco_hyemalis	Passeriformes	7.60	8.95	0.83	19.50	So	Mi	Pl	Ge	D
Jynx_torquilla	Piciformes	7.50	9.43	0.94	34.96	De	Mi	An	Te	D
Kaupifalco_monogrammicus	Accipitriformes	19.30	NA	4.03	278.94	Op	Se	An	In	D
Ketupa_ketupu	Strigiformes	32.10	NA	12.75	1293.00	So	Se	An	In	N
Lagonosticta_senegala	Passeriformes	4.50	6.05	0.39	9.21	So	Se	Pl	Te	D
Lagopus_lagopus	Galliformes	12.80	15.80	2.38	566.86	Op	Se	Pl	Te	D

Lagopus_muta	Galliformes	12.40	15.55	2.50	535.30	Op	Se	Pl	Te	D
Lamprotornis_superbus	Passeriformes	10.00	12.30	1.72	64.90	So	Se	An	Te	D
Lanius_collaris	Passeriformes	8.50	10.90	1.07	36.44	Op	Se	An	In	D
Lanius_collurio	Passeriformes	9.00	11.00	0.96	28.44	Op	Mi	An	In	D
Lanius_cristatus	Passeriformes	9.30	11.90	1.05	32.18	Op	Mi	An	In	D
Lanius_excubitor	Passeriformes	8.78	10.04	1.43	63.41	Op	Mi	An	In	D
Lanius_ludovicianus	Passeriformes	11.20	13.05	1.53	51.59	Op	Mi	An	In	D
Lanius_senator	Passeriformes	9.00	11.40	1.07	35.97	So	Mi	An	In	D
Lanius_tigrinus	Passeriformes	9.90	11.80	1.10	30.14	So	Mi	An	In	D
Lanius_vittatus	Passeriformes	9.00	10.40	0.82	20.80	So	Pm	An	In	D
Larus_argentatus	Charadriiformes	18.70	NA	6.69	1090.99	Op	Pm	An	Ge	D
Larus_canus	Charadriiformes	17.40	NA	4.07	412.53	Op	Mi	An	Te	D
Larus_delawarensis	Charadriiformes	17.23	NA	4.44	516.31	Op	Pm	An	Te	D
Larus_marinus	Charadriiformes	19.80	NA	8.20	1649.71	Op	Pm	Om	Te	D
Larus_philadelphia	Charadriiformes	12.25	NA	2.26	210.71	Op	Pm	An	Ae	D
Larus_ridibundus	Charadriiformes	11.50	NA	3.05	284.00	Op	Pm	An	Ge	D
Lathamus_discolor	Psittaciformes	8.05	9.73	2.37	64.70	So	Mi	Pl	In	D
Legatus_leucophaius	Passeriformes	7.90	9.75	0.66	22.20	De	Se	Pl	In	D
Leiothrix_argentauris	Passeriformes	8.00	9.50	0.94	28.40	De	Se	An	In	D
Leiothrix_lutea	Passeriformes	8.30	9.50	0.92	21.39	De	Se	An	In	D
Lepidothrix_coronata	Passeriformes	6.00	7.30	0.36	8.34	De	Se	Pl	In	D
Leptosomus_discolor	Leptosomiformes	18.90	22.61	4.11	255.00	De	Se	An	In	D
Leptotila_cassini	Columbiformes	10.50	13.35	1.30	159.00	De	Se	Pl	Te	D
Leptotila_rufaxilla	Columbiformes	9.80	13.15	1.27	157.00	De	Se	Pl	Te	D
Leptotila_verreauxi	Columbiformes	11.60	13.73	1.42	146.88	De	Se	Pl	Te	D
Lessonia_rufa	Passeriformes	6.70	7.80	0.43	13.40	Op	Mi	An	Te	D
Lichenostomus_ornatus	Passeriformes	6.50	7.95	0.75	17.63	So	Se	Om	In	D
Lichenostomus_unicolor	Passeriformes	7.20	8.75	0.82	30.70	So	Se	Pl	In	D
Lichmera_indistincta	Passeriformes	5.20	6.15	0.51	11.18	So	Se	Pl	In	D
Limicola_falcinellus	Charadriiformes	6.50	NA	0.72	37.10	Op	Mi	An	Te	D
Limnodromus_griseus	Charadriiformes	8.80	NA	1.38	110.53	Op	Mi	An	Te	D
Limnodromus_scolopaceus	Charadriiformes	8.30	NA	1.37	104.40	Op	Mi	An	Te	D

Limnothlypis_swainsonii	Passeriformes	6.30	7.65	0.67	18.90	De	Mi	An	Te	D
Limosa_lapponica	Charadriiformes	9.40	NA	2.77	291.65	Op	Mi	An	Te	D
Limosa_limosa	Charadriiformes	9.80	NA	2.70	288.37	Op	Mi	An	Te	D
Lipaugus_vociferans	Passeriformes	12.10	14.40	1.63	75.42	De	Se	Om	In	D
Lonchura_bicolor	Passeriformes	4.75	5.50	0.42	9.90	So	Se	Pl	In	D
Lonchura_punctulata	Passeriformes	5.20	6.25	0.46	13.60	So	Se	Pl	Ge	D
Lonchura_striata	Passeriformes	5.50	6.80	0.74	12.30	Op	Se	Pl	In	D
Lophophorus_impejanus	Galliformes	9.33	11.75	6.20	2066.51	Op	Se	Pl	Te	D
Lophorina_superba	Passeriformes	10.20	12.10	2.60	76.66	De	Se	Om	In	D
Lophura_swinhoii	Galliformes	7.00	9.80	4.25	1100.00	De	Se	Pl	Te	D
Lorius_garrulus	Psittaciformes	10.20	12.70	5.17	143.00	De	Se	Pl	In	D
Lorius_lory	Psittaciformes	10.30	12.65	5.12	176.89	De	Se	Pl	In	D
Loxia_leucoptera	Passeriformes	6.40	7.25	1.38	28.69	De	Mi	Pl	In	D
Loxigilla_violacea	Passeriformes	7.55	9.45	1.16	22.17	So	Se	Pl	In	D
Lullula_arborea	Passeriformes	7.80	9.40	0.82	26.90	Op	Pm	Om	Te	D
Luscinia_megarhynchos	Passeriformes	7.60	9.20	0.70	19.60	De	Mi	An	Ge	D
Machetornis_rixosa	Passeriformes	8.70	10.15	0.96	29.60	Op	Se	An	Te	D
Macronous_gularis	Passeriformes	6.40	7.40	0.65	11.83	So	Se	An	In	D
Macropygia_phasianella	Columbiformes	9.40	11.95	1.46	237.00	De	Pm	Pl	In	D
Macropygia_unchall	Columbiformes	10.80	13.30	1.48	168.00	De	Se	Pl	In	D
Malaconotus_cruentus	Passeriformes	11.85	14.60	2.28	76.69	De	Se	An	In	D
Malacoptila_panamensis	Piciformes	10.70	13.60	1.15	42.60	De	Se	An	In	D
Malurus_cyaneus	Passeriformes	6.00	7.35	0.47	10.33	So	Se	An	Te	D
Malurus_lamberti	Passeriformes	6.30	7.45	0.46	8.04	So	Se	An	In	D
Malurus_melanocephalus	Passeriformes	5.70	7.05	0.45	7.90	De	Se	An	Te	D
Manacus_manacus	Passeriformes	7.25	8.58	0.59	16.70	De	Se	Pl	In	D
Manacus_vitellinus	Passeriformes	7.10	8.55	0.62	18.16	De	Se	Pl	In	D
Manorina_flavigula	Passeriformes	8.80	10.95	1.37	55.90	So	Se	An	In	D
Manorina_melanophrys	Passeriformes	7.20	8.80	1.14	30.39	So	Se	An	In	D
Margarops_fuscatus	Passeriformes	11.30	13.35	2.35	98.43	So	Se	Om	In	D
Margarops_fuscus	Passeriformes	10.30	12.10	1.54	67.07	De	Se	Om	In	D
Mecocerculus_leucophrys	Passeriformes	6.60	7.80	0.48	10.98	De	Se	An	In	D

Megaceryle_alcyon	Coraciiformes	13.50	16.33	2.46	148.00	Op	Pm	An	In	D
Megaceryle_torquata	Coraciiformes	16.60	20.20	3.57	317.00	Op	Se	An	In	D
Megalaima_asiatica	Piciformes	10.90	13.10	1.53	90.50	De	Se	Pl	In	D
Megalaima_haemacephala	Piciformes	8.90	11.45	0.97	44.49	So	Se	Om	In	D
Megalaima_oorti	Piciformes	10.70	12.70	1.45	87.69	De	Se	Pl	In	D
Megalaima_virens	Piciformes	12.60	15.35	2.12	202.00	De	Se	Pl	In	D
Megalaima_zeylanica	Piciformes	11.90	14.65	1.90	119.00	De	Se	Pl	In	D
Megapodius_freycinet	Galliformes	12.23	15.50	3.78	642.48	So	Se	Pl	Te	D
Megarynchus_pitangua	Passeriformes	10.65	12.75	1.17	69.91	So	Se	An	In	D
Melampitta_lugubris	Passeriformes	11.70	13.40	2.10	42.67	De	Se	An	Te	D
Melanerpes_aurifrons	Piciformes	9.50	11.70	2.20	80.77	So	Se	Om	In	D
Melanerpes_carolinus	Piciformes	9.80	11.95	2.16	69.50	De	Se	Om	In	D
Melanerpes_erythrocephalus	Piciformes	9.00	10.83	1.78	71.60	De	Se	An	In	D
Melanerpes_formicivorus	Piciformes	9.10	11.75	1.99	79.62	So	Se	Pl	In	D
Melanerpes_lewis	Piciformes	9.90	12.85	2.22	105.76	So	Mi	Om	In	D
Melanocharis_nigra	Passeriformes	6.70	8.00	0.54	14.16	De	Se	Om	In	D
Melanodryas_cucullata	Passeriformes	8.50	10.30	0.80	21.10	So	Se	An	In	D
Melanoptila_glabrirostris	Passeriformes	9.30	11.20	1.20	35.40	So	Se	Om	In	D
Meleagris_gallopavo	Galliformes	17.50	21.17	8.21	5791.37	So	Se	Pl	Te	D
Meleagris_ocellata	Galliformes	19.20	23.60	5.84	5525.00	So	Se	Pl	Te	D
Melidectes_torquatus	Passeriformes	8.30	10.60	1.45	44.93	So	Se	Om	In	D
Melidora_macrorrhina	Coraciiformes	15.20	18.18	2.30	97.00	De	Se	An	In	N
Melilestes_megarhynchus	Passeriformes	8.20	9.80	1.27	43.76	De	Se	Om	In	D
Meliphaga_lewinii	Passeriformes	7.80	9.90	1.17	33.58	De	Se	Pl	In	D
Melipotes_fumigatus	Passeriformes	9.10	11.50	1.55	58.60	De	Se	Pl	In	D
Melithreptus_brevirostris	Passeriformes	5.60	6.85	0.68	12.60	So	Se	Om	In	D
Melithreptus_lunatus	Passeriformes	5.40	7.40	0.60	14.01	So	Se	An	In	D
Mellisuga_minima	Caprimulgiformes	3.40	4.50	0.13	2.40	So	Se	Pl	Ae	D
Melopsittacus_undulatus	Psittaciformes	6.90	7.50	1.50	28.99	Op	Pm	Pl	Ge	D
Melospiza_georgiana	Passeriformes	6.90	7.95	0.81	16.10	So	Mi	Om	Ge	D
Melospiza_melodia	Passeriformes	6.80	8.45	1.06	21.91	So	Pm	Om	Ge	D
Menura_novaehollandiae	Passeriformes	17.60	22.28	10.71	972.62	De	Se	An	Te	D

Mergus_serrator	Anseriformes	11.73	NA	4.83	1015.17	Op	Mi	An	Aq	D
Merops_albicollis	Coraciiformes	8.00	9.80	0.51	25.90	Op	Pm	An	In	D
Merops_nubicus	Coraciiformes	9.60	11.80	0.87	42.40	So	Pm	An	In	D
Merops_orientalis	Coraciiformes	7.20	8.65	0.48	14.80	So	Se	An	In	D
Merops_pusillus	Coraciiformes	6.40	8.00	0.41	15.10	So	Se	An	In	D
Microeca_fascinans	Passeriformes	6.90	8.40	0.54	14.47	So	Mi	An	In	D
Microeca_flavigaster	Passeriformes	7.00	8.70	0.51	12.10	So	Se	An	In	D
Microhierax_erythrogenys	Falconiformes	10.95	NA	1.40	49.96	So	Se	An	In	D
Microhierax_fringillarius	Falconiformes	9.50	NA	1.16	42.90	So	Se	An	In	D
Micropsitta_pusio	Psittaciformes	5.70	7.25	0.98	14.00	So	Se	Pl	In	D
Milvus_migrans	Accipitriformes	20.00	NA	5.82	734.10	Op	Mi	Om	Ae	D
Milvus_milvus	Accipitriformes	20.30	NA	7.72	1071.77	So	Pm	Om	Ge	D
Mimus_polyglottos	Passeriformes	8.90	11.70	1.38	48.50	So	Mi	Om	Ge	D
Minla_ignotincta	Passeriformes	6.40	7.50	0.71	14.30	So	Se	An	In	D
Mino_dumontii	Passeriformes	12.30	14.60	2.98	217.00	De	Se	Pl	In	D
Mionectes_oleagineus	Passeriformes	6.57	7.60	0.45	11.17	De	Se	Pl	In	D
Mirafra_javanica	Passeriformes	6.00	8.60	0.73	23.00	Op	Se	Om	Te	D
Mitrephanes_phaeocercus	Passeriformes	6.20	7.50	0.32	8.59	De	Se	An	In	D
Mitrospingus_cassinii	Passeriformes	8.40	10.35	1.31	40.40	So	Se	Om	In	D
Mniotilta_varia	Passeriformes	5.80	7.10	0.44	10.90	De	Mi	An	In	D
Molothrus_ater	Passeriformes	8.20	9.15	1.15	40.26	So	Mi	An	Te	D
Molothrus_oryzivorus	Passeriformes	11.70	13.30	3.14	181.85	De	Se	Om	Te	D
Momotus_momota	Coraciiformes	14.00	17.65	2.19	114.96	De	Se	An	In	D
Monachella_muelleriana	Passeriformes	7.90	9.60	0.75	24.50	Op	Se	An	In	D
Monarcha_trivirgatus	Passeriformes	7.70	9.50	0.63	12.70	De	Se	An	In	D
Monias_benschi	Mesitornithiformes	10.10	12.45	2.00	137.94	De	Se	An	Te	D
Monticola_solitarius	Passeriformes	9.40	13.30	1.27	53.65	Op	Pm	An	In	D
Montifringilla_nivalis	Passeriformes	6.70	8.55	1.05	36.90	Op	Pm	Pl	Te	D
Motacilla_alba	Passeriformes	7.10	8.25	0.60	23.93	Op	Mi	An	Te	D
Motacilla_cinerea	Passeriformes	6.20	7.75	0.51	17.15	Op	Pm	An	Te	D
Motacilla_flava	Passeriformes	7.20	8.60	0.55	17.68	Op	Mi	An	Te	D
Mulleripicus_fulvus	Piciformes	12.80	14.55	4.43	312.25	De	Se	An	In	D

Muscisaxicola_alpinus	Passeriformes	7.30	9.80	0.78	26.10	Op	Pm	An	Te	D
Musophaga_rossae	Musophagiformes	14.90	17.75	4.05	361.42	So	Se	Pl	In	D
Musophaga_violacea	Musophagiformes	13.80	17.15	3.57	360.00	So	Se	Pl	In	D
Myiagra_inquieta	Passeriformes	7.90	9.50	0.76	20.90	So	Pm	An	In	D
Myiagra_rubecula	Passeriformes	7.30	8.90	0.54	12.05	So	Se	An	In	D
Myiarchus_cinerascens	Passeriformes	9.10	10.50	0.77	28.20	So	Mi	An	In	D
Myiarchus_crinitus	Passeriformes	8.38	8.01	0.91	32.10	De	Mi	Om	In	D
Myiarchus_stolidus	Passeriformes	8.60	10.35	0.65	20.80	De	Se	An	In	D
Myiarchus_tuberculifer	Passeriformes	8.50	9.80	0.66	17.70	De	Se	An	In	D
Myiarchus_tyrannulus	Passeriformes	9.10	11.03	0.98	35.45	So	Pm	An	In	D
Myiobius_barbatus	Passeriformes	6.90	8.70	0.35	11.00	De	Se	An	In	D
Myiodynastes_maculatus	Passeriformes	9.73	11.84	1.13	43.20	So	Mi	An	In	D
Myiophobus_fasciatus	Passeriformes	6.70	8.15	0.37	9.90	So	Mi	An	In	D
Myiopsitta_monachus	Psittaciformes	8.70	10.20	4.08	120.00	So	Se	Pl	In	D
Myiozetetes_similis	Passeriformes	8.00	10.05	0.82	28.00	So	Pm	An	In	D
Myrmeciza_exsul	Passeriformes	9.80	10.40	1.03	26.50	De	Se	An	In	D
Myrmeciza_ferruginea	Passeriformes	6.60	9.30	0.82	26.10	De	Se	An	Te	D
Myrmotherula_axillaris	Passeriformes	5.30	6.90	0.39	8.09	De	Se	An	In	D
Myzomela_obscura	Passeriformes	5.70	6.35	0.51	11.88	So	Se	Pl	In	D
Nandayus_nenday	Psittaciformes	9.90	11.65	4.55	128.00	So	Se	Pl	In	D
Necrosyrtes_monachus	Accipitriformes	21.00	NA	12.25	2043.00	So	Se	Ca/Re	Te	D
Nectarinia_jugularis	Passeriformes	4.80	6.45	0.40	8.99	So	Se	Om	In	D
Nectarinia_olivacea	Passeriformes	6.00	7.40	0.46	9.65	De	Se	Om	In	D
Neochmia_phaeton	Passeriformes	5.50	6.85	0.50	10.00	So	Se	Pl	Ge	D
Neochmia_temporalis	Passeriformes	4.80	6.00	0.52	11.40	So	Se	Pl	Te	D
Neophema_splendida	Psittaciformes	6.80	NA	1.28	38.60	So	Se	Pl	Te	D
Neophron_percnopterus	Accipitriformes	22.05	NA	10.62	2082.00	Op	Mi	Om	Ge	D
Neopsephotus_bourkii	Psittaciformes	6.63	8.54	1.25	44.40	So	Se	Pl	Te	D
Neopsittacus_musschenbroekii	Psittaciformes	7.60	8.90	2.45	50.90	De	Se	Pl	In	D
Neotis_denhami	Otidiformes	18.10	21.20	12.25	3970.20	Op	Se	An	Te	D
Nestor_meridionalis	Psittaciformes	12.70	14.70	13.00	428.01	De	Se	Pl	In	D
Nestor_notabilis	Psittaciformes	14.30	16.68	14.40	862.97	So	Se	Pl	In	D

Nicator_chloris	Passeriformes	9.80	12.20	1.28	46.10	De	Se	An	In	D
Niltava_grandis	Passeriformes	10.80	13.35	0.95	30.30	De	Se	An	In	D
Ninox_connivens	Strigiformes	25.30	NA	6.40	577.99	So	Se	An	In	N
Ninox_jacquinoti	Strigiformes	19.10	NA	4.38	200.81	De	Se	An	In	N
Ninox_novaeseelandiae	Strigiformes	22.30	NA	5.53	183.37	De	Pm	An	In	N
Notharchus_macrorhynchos	Piciformes	15.20	17.48	1.70	95.89	So	Se	An	In	D
Nothoprocta_ornata	Struthioniformes	12.10	15.90	2.85	619.27	So	Se	Pl	Te	D
Nothoprocta_perdicaria	Struthioniformes	10.23	12.04	1.88	458.00	So	Se	Pl	Te	D
Nothura_maculosa	Struthioniformes	11.00	13.85	1.58	257.48	So	Se	Pl	Te	D
Nucifraga_caryocatactes	Passeriformes	14.00	17.20	5.32	182.51	De	Pm	Om	In	D
Numenius_arquata	Charadriiformes	14.70	NA	4.63	802.99	Op	Mi	An	Te	D
Numenius_phaeopus	Charadriiformes	13.40	NA	3.13	364.57	Op	Mi	An	Te	D
Numenius_tahitiensis	Charadriiformes	13.10	NA	3.42	400.08	Op	Mi	Om	Te	D
Numida_meleagris	Galliformes	14.30	18.70	4.08	1299.00	So	Se	Pl	Te	D
Nyctibius_griseus	Caprimulgiformes	20.40	NA	1.98	172.04	De	Se	An	In	N
Nyctidromus_albicollis	Caprimulgiformes	13.40	NA	0.91	57.84	So	Se	An	In	N
Nyctiphrynus_ocellatus	Caprimulgiformes	10.33	NA	0.74	39.60	De	Se	An	In	N
Nyctiprogne_leucopyga	Caprimulgiformes	9.80	NA	0.51	27.30	De	Se	An	Ae	N
Nymphicus_hollandicus	Psittaciformes	7.54	9.19	2.39	94.61	Op	Pm	Pl	Te	D
Ocyphaps_lophotes	Columbiformes	11.20	13.25	1.70	204.00	Op	Se	Pl	Te	D
Oena_capensis	Columbiformes	7.80	9.25	0.61	40.60	Op	Pm	Pl	Te	D
Oenanthe_oenanthe	Passeriformes	7.20	9.70	0.78	25.39	Op	Mi	An	In	D
Onychorhynchus_coronatus	Passeriformes	8.15	9.43	0.47	14.00	De	Pm	An	In	D
Opisthocomus_hoazin	Opisthocomiformes	10.73	13.18	3.89	696.00	So	Se	Pl	In	D
Oreocharis_arfaki	Passeriformes	7.20	8.20	0.68	19.70	De	Se	Pl	In	D
Oreoica_gutturalis	Passeriformes	9.60	12.25	1.67	62.80	So	Se	An	Te	D
Oreopsittacus_arfaki	Psittaciformes	5.80	6.93	1.15	20.20	De	Se	Pl	In	D
Oreortyx_pictus	Galliformes	10.10	12.95	1.68	233.00	So	Se	Pl	Te	D
Origma_solitaria	Passeriformes	6.80	8.40	0.85	14.34	Op	Se	An	Ge	D
Oriolus_chinensis	Passeriformes	11.26	13.73	1.80	82.60	So	Pm	Om	In	D
Oriolus_flavocinctus	Passeriformes	11.30	13.50	2.10	109.00	De	Se	Pl	In	D
Oriolus_oriolus	Passeriformes	10.35	12.53	1.45	79.00	De	Mi	Om	In	D

Oriolus_sagittatus	Passeriformes	11.20	14.05	1.96	95.20	So	Pm	Pl	In	D
Ortalis_vetula	Galliformes	14.90	18.80	3.23	562.60	De	Se	Pl	In	D
Orthonyx_spaldingii	Passeriformes	11.40	13.30	2.90	149.91	De	Se	An	Te	D
Orthonyx_temminckii	Passeriformes	8.00	9.88	1.76	58.06	De	Se	An	Te	D
Orthorhyncus_cristatus	Caprimulgiformes	3.50	4.70	0.14	3.02	So	Se	Pl	Ae	D
Ortygospiza_atricollis	Passeriformes	3.70	5.85	0.34	10.60	Op	Se	Pl	Te	D
Oryzoborus_angolensis	Passeriformes	6.20	7.35	0.68	13.00	So	Se	Om	In	D
Otidiphaps_nobilis	Columbiformes	14.00	17.45	2.60	499.99	So	Pm	Pl	Te	D
Otis_tarda	Otidiformes	27.00	31.25	8.46	6759.92	Op	Pm	Om	Te	D
Otus_scops	Strigiformes	14.70	NA	2.49	92.00	So	Mi	An	In	N
Pachycephala_olivacea	Passeriformes	10.60	12.25	1.48	41.20	De	Se	An	In	D
Pachycephala_pectoralis	Passeriformes	10.60	12.90	1.12	30.36	So	Pm	An	In	D
Pachycephala_rufiventris	Passeriformes	9.00	10.35	1.03	22.48	So	Se	An	In	D
Pachycephala_simplex	Passeriformes	7.80	9.80	0.83	17.40	De	Se	An	In	D
Pachycephalopsis_poliosoma	Passeriformes	10.20	12.30	1.06	37.97	De	Se	An	In	D
Pachyramphus_cinnamomeus	Passeriformes	9.20	10.50	0.88	20.30	De	Se	An	In	D
Pachyramphus_polychopterus	Passeriformes	9.80	11.25	0.74	20.80	So	Se	Om	In	D
Padda_oryzivora	Passeriformes	5.80	7.50	0.85	24.80	Op	Se	Pl	Ge	D
Panurus_biarmicus	Passeriformes	5.10	6.90	0.55	13.82	Op	Pm	Om	In	D
Pardalotus_striatus	Passeriformes	5.80	7.00	0.52	11.44	So	Se	An	In	D
Paroaria_dominicana	Passeriformes	7.00	8.80	1.12	33.20	So	Se	Om	Te	D
Parotia_lawesii	Passeriformes	13.90	16.35	4.01	161.40	De	Se	Om	In	D
Parus_atricapillus	Passeriformes	6.30	7.10	0.76	10.80	De	Se	Om	In	D
Parus_caeruleus	Passeriformes	5.30	6.50	0.67	11.10	De	Se	Om	In	D
Parus_gambeli	Passeriformes	6.00	7.30	0.75	11.19	De	Se	Om	In	D
Parus_major	Passeriformes	6.60	NA	0.83	16.25	So	Se	Om	In	D
Parus_palustris	Passeriformes	6.00	6.85	0.56	11.14	De	Se	Om	In	D
Passer_domesticus	Passeriformes	5.54	6.13	0.97	26.51	Op	Se	Pl	Te	D
Passer_griseus	Passeriformes	7.20	8.50	0.94	27.82	Op	Se	Pl	Te	D
Passer_hispaniolensis	Passeriformes	6.30	7.50	0.97	24.20	So	Se	Pl	Te	D
Passer_rutilans	Passeriformes	6.30	7.33	0.66	18.46	So	Se	Pl	Te	D
Passerculus_sandwichensis	Passeriformes	6.50	7.55	0.68	19.97	Op	Mi	Om	Ge	D

Passerina_cyanea	Passeriformes	6.20	7.39	0.65	14.69	So	Mi	Pl	Te	D
Patagioenas_cayennensis	Columbiformes	11.35	14.68	1.76	229.00	Op	Se	Pl	In	D
Patagioenas_leucocephala	Columbiformes	10.30	14.05	1.74	240.14	So	Se	Pl	In	D
Pavo_cristatus	Galliformes	17.50	22.30	6.86	4154.81	So	Se	Om	Te	D
Pavo_muticus	Galliformes	20.80	23.45	6.27	2205.71	So	Se	An	Te	D
Pedionomus_torquatus	Charadriiformes	8.50	10.73	0.77	62.52	Op	Se	Om	Te	D
Pelargopsis_capensis	Coraciiformes	14.30	16.60	2.82	178.68	So	Pm	An	In	D
Pelecanus_occidentalis	Pelecaniformes	19.52	NA	19.68	3427.84	Op	Se	An	Ae	D
Penelopides_panini	Bucerotiformes	16.80	20.55	6.88	470.54	De	Se	Pl	In	D
Peneothello_cyanus	Passeriformes	10.10	12.25	0.96	24.98	De	Se	An	In	D
Pericrocotus_solaris	Passeriformes	9.30	10.55	0.73	14.50	So	Se	An	In	D
Perisoreus_canadensis	Passeriformes	12.60	14.15	2.53	71.58	De	Se	Om	In	D
Perissocephalus_tricolor	Passeriformes	17.40	21.08	4.70	338.88	De	Se	Pl	In	D
Pernis_apivorus	Accipitriformes	21.50	NA	6.95	754.37	De	Mi	An	In	D
Petrochelidon_pyrrhonota	Passeriformes	8.60	9.55	0.49	21.60	Op	Mi	An	Ae	D
Petroica_multicolor	Passeriformes	7.30	8.70	0.52	11.36	De	Se	An	In	D
Petroica_phoenicea	Passeriformes	6.90	8.70	0.49	13.00	So	Mi	An	In	D
Petroica_rosea	Passeriformes	6.70	8.35	0.43	8.20	De	Mi	An	In	D
Pezoporus_wallicus	Psittaciformes	11.00	12.60	2.66	71.21	So	Se	Pl	Ge	D
Phacellodomus_ruber	Passeriformes	7.00	8.20	1.11	41.00	So	Se	An	Ge	D
Phaenicophaeus_chlorophaeus	Cuculiformes	8.30	10.80	1.25	51.21	So	Se	An	In	D
Phaenicophaeus_curvirostris	Cuculiformes	15.80	18.40	2.92	154.00	De	Se	An	In	D
Phaenicophaeus_superciliosus	Cuculiformes	12.40	15.25	2.51	123.00	So	Se	An	In	D
Phaenicophaeus_tristis	Cuculiformes	13.50	15.98	2.30	117.00	De	Se	An	In	D
Phaenicophilus_palmarum	Passeriformes	8.00	9.85	1.13	30.50	So	Se	An	In	D
Phaenostictus_mcleannani	Passeriformes	8.90	10.95	1.07	51.10	De	Se	An	In	D
Phaeomyias_murina	Passeriformes	7.10	8.10	0.37	10.00	So	Se	Om	In	D
Phaeothlypis_fulvicauda	Passeriformes	6.50	8.40	0.56	14.90	De	Se	An	Te	D
Phaethornis_superciliosus	Caprimulgiformes	4.50	5.85	0.26	6.30	De	Se	Pl	Ae	D
Phainopepla_nitens	Passeriformes	7.70	8.88	0.79	22.10	So	Se	Om	In	D
Phalacrocorax_carbo	Suliformes	15.00	NA	10.45	2528.97	Op	Pm	An	Aq	D
Phalaenoptilus_nuttallii	Caprimulgiformes	10.80	NA	0.53	48.33	Op	Pm	An	Ge	N

Phalaropus_fulicarius	Charadriiformes	6.95	NA	0.56	55.59	Op	Mi	An	Te	D
Phalaropus_lobatus	Charadriiformes	6.00	NA	0.45	36.68	Op	Mi	An	Te	D
Pharomachrus_mocinno	Trogoniformes	11.82	13.01	2.34	202.46	De	Se	Pl	In	D
Phasianus_colchicus	Galliformes	10.80	13.71	3.86	1120.31	So	Se	Pl	Te	D
Pheucticus_ludovicianus	Passeriformes	9.15	10.90	1.32	42.00	So	Mi	Pl	In	D
Phigys_solitarius	Psittaciformes	8.20	10.20	2.75	83.26	De	Se	Pl	In	D
Philemon_citreogularis	Passeriformes	9.00	11.15	1.58	60.56	So	Pm	Pl	In	D
Philemon_corniculatus	Passeriformes	10.50	12.35	1.98	100.71	So	Se	Om	In	D
Philomachus_pugnax	Charadriiformes	10.10	NA	1.56	131.68	Op	Mi	An	Te	D
Phleocryptes_melanops	Passeriformes	6.80	7.80	0.69	14.60	So	Se	An	In	D
Phoenicopterus_ruber	Phoenicopteriformes	14.00	NA	10.80	3031.59	Op	Se	An	Te	D
Phoeniculus_purpureus	Bucerotiformes	8.83	10.60	1.95	73.21	So	Se	An	In	D
Phoenicurus_phoenicurus	Passeriformes	7.60	8.90	0.51	14.59	De	Mi	An	In	D
Phylidonyris_niger	Passeriformes	6.80	8.25	0.80	18.20	So	Pm	Pl	In	D
Phylidonyris_novaehollandiae	Passeriformes	6.10	8.00	0.90	20.21	So	Pm	Pl	In	D
Phylloscopus_collybita	Passeriformes	5.40	6.50	0.31	8.30	De	Mi	An	In	D
Phylloscopus_trochilus	Passeriformes	5.50	7.10	0.34	8.70	De	Mi	An	In	D
Phytotoma_rara	Passeriformes	8.58	10.41	1.16	47.00	So	Se	Pl	In	D
Phytotoma_rutila	Passeriformes	7.93	9.63	0.73	40.50	So	Se	Pl	In	D
Piaya_cayana	Cuculiformes	12.60	14.70	1.84	101.98	De	Se	An	In	D
Pica_pica	Passeriformes	14.80	16.20	4.67	217.48	Op	Se	Om	Te	D
Picoides_arcticus	Piciformes	8.80	11.55	3.15	69.24	De	Se	An	In	D
Picoides_pubescens	Piciformes	6.70	9.08	1.21	25.55	So	Se	An	In	D
Picoides_villosus	Piciformes	6.80	8.90	2.95	62.65	De	Se	An	In	D
Piculus_flavigula	Piciformes	8.00	9.85	1.63	55.00	De	Se	An	In	D
Picumnus_innominatus	Piciformes	5.20	6.80	0.55	10.20	De	Se	An	In	D
Picumnus_temminckii	Piciformes	5.10	6.55	0.60	11.50	So	Se	An	In	D
Picus_viridis	Piciformes	11.80	14.40	4.24	176.00	So	Se	An	Te	D
Pinicola_enucleator	Passeriformes	8.70	10.20	1.58	56.40	De	Pm	Pl	In	D
Pionites_melanocephalus	Psittaciformes	11.80	14.10	5.18	148.78	De	Se	Pl	In	D
Pionopsitta_pileata	Psittaciformes	9.80	11.55	2.77	119.00	De	Se	Pl	In	D
Pionus_menstruus	Psittaciformes	12.50	15.80	5.70	251.00	So	Se	Pl	In	D

Pipile_pipile	Galliformes	16.80	20.10	5.30	1816.59	De	Se	Pl	In	D
Pipilo_erythrophthalmus	Passeriformes	8.10	10.20	1.36	40.03	So	Pm	Pl	Ge	D
Pipilo_fuscus	Passeriformes	8.80	10.30	1.55	44.40	So	Se	Pl	Ge	D
Pipra_erythrocephala	Passeriformes	7.60	8.45	0.49	13.43	De	Se	Pl	In	D
Pipra_fasciicauda	Passeriformes	7.30	9.28	0.62	15.90	De	Se	Pl	In	D
Pipra_pipra	Passeriformes	7.00	8.55	0.50	11.11	De	Se	Om	In	D
Pitangus_sulphuratus	Passeriformes	9.55	11.90	1.35	62.85	Op	Se	An	In	D
Pithecophaga_jefferyi	Accipitriformes	19.00	NA	19.75	5175.32	De	Se	An	In	D
Pithys_albifrons	Passeriformes	7.50	8.70	0.62	19.47	De	Se	An	In	D
Pitohui_ferrugineus	Passeriformes	11.40	14.50	2.48	93.91	De	Se	Om	In	D
Pitta_brachyura	Passeriformes	11.40	13.80	1.39	55.50	De	Mi	An	Te	D
Pitta_guajana	Passeriformes	11.80	14.15	1.37	81.50	De	Se	An	Te	D
Pitta_maxima	Passeriformes	14.80	18.30	2.53	190.00	De	Se	An	Te	D
Pitta_sordida	Passeriformes	11.30	13.10	1.31	64.50	De	Mi	An	Te	D
Pitta_versicolor	Passeriformes	12.00	14.60	1.87	88.94	De	Se	An	Te	D
Platalea_leucorodia	Pelecaniformes	13.90	NA	10.42	1868.00	Op	Pm	An	Te	D
Platycercus_adscitus	Psittaciformes	9.60	10.90	3.06	111.00	So	Se	Pl	In	D
Platyrinchus_mystaceus	Passeriformes	7.77	9.00	0.40	9.70	De	Se	An	In	D
Platysteira_cyanea	Passeriformes	7.95	9.28	0.79	14.60	So	Se	An	In	D
Plectorhyncha_lanceolata	Passeriformes	7.30	9.20	1.09	40.56	So	Se	Pl	In	D
Plectrophenax_nivalis	Passeriformes	7.40	8.80	1.02	42.20	Op	Mi	Pl	Te	D
Plegadis_falcinellus	Pelecaniformes	11.40	NA	5.12	626.61	Op	Pm	An	Te	D
Ploceus_cucullatus	Passeriformes	8.00	9.70	1.37	36.08	Op	Se	Pl	In	D
Pluvialis_apricaria	Charadriiformes	15.40	NA	2.23	214.00	Op	Mi	An	Te	D
Pluvialis_dominica	Charadriiformes	11.80	NA	1.82	151.47	Op	Mi	An	Te	D
Pluvialis_squatarola	Charadriiformes	12.80	NA	2.56	250.00	Op	Mi	An	Te	D
Pluvianellus_socialis	Charadriiformes	8.80	NA	1.14	84.11	Op	Pm	An	Te	D
Pluvianus_aegyptius	Charadriiformes	9.80	12.30	1.28	82.00	Op	Pm	An	Te	D
Podager_nacunda	Caprimulgiformes	15.42	NA	1.29	159.00	So	Se	An	Ae	D
Podargus_strigoides	Caprimulgiformes	24.57	NA	4.86	308.03	De	Se	An	In	N
Podiceps_cristatus	Podicipediformes	13.00	NA	3.11	730.96	Op	Se	An	Aq	D
Poecilodryas_superciliosa	Passeriformes	8.60	10.15	0.92	19.80	So	Se	An	In	D

Poeoptera_lugubris	Passeriformes	7.10	8.90	0.99	38.79	De	Se	Pl	In	D
Poephila_acuticauda	Passeriformes	5.90	6.65	0.51	14.00	Op	Pm	Pl	Ge	D
Poephila_cincta	Passeriformes	5.80	7.15	0.47	14.90	So	Pm	Pl	Te	D
Pogoniulus_scolopaceus	Piciformes	7.13	8.42	0.44	14.07	De	Se	Om	In	D
Poicephalus_meyeri	Psittaciformes	10.70	12.85	4.41	117.47	So	Se	Pl	In	D
Poicephalus_senegalus	Psittaciformes	10.70	13.45	4.71	147.00	Op	Se	Pl	In	D
Polioptila_caerulea	Passeriformes	5.30	6.80	0.30	5.80	So	Mi	An	In	D
Polioptila_dumicola	Passeriformes	5.80	6.90	0.42	7.00	So	Se	An	In	D
Polyboroides_typus	Accipitriformes	16.40	NA	8.00	633.24	De	Se	An	In	D
Polytelis_alexandrae	Psittaciformes	8.65	10.83	2.58	103.69	Op	Pm	Pl	In	D
Pomatorhinus_ruficollis	Passeriformes	7.80	9.45	1.53	31.61	So	Se	An	Ge	D
Pomatostomus_temporalis	Passeriformes	8.30	9.68	1.90	74.99	So	Se	An	Ge	D
Poospiza_nigrorufa	Passeriformes	6.60	7.95	0.70	17.40	So	Se	Om	Ge	D
Prionops_plumatus	Passeriformes	9.35	11.15	1.16	33.78	So	Se	An	In	D
Priotelus_roseigaster	Trogoniformes	11.30	13.95	1.31	74.19	De	Se	An	In	D
Priotelus_temnurus	Trogoniformes	12.00	13.85	1.16	53.40	De	Se	Pl	In	D
Probosciger_aterrimus	Psittaciformes	17.60	21.05	20.50	841.00	So	Se	Pl	In	D
Procnias_nudicollis	Passeriformes	13.50	16.15	2.70	172.04	De	Se	Pl	In	D
Progne_subis	Passeriformes	9.20	11.38	1.03	53.79	So	Se	An	Ae	D
Prosopeia_tabuensis	Psittaciformes	13.10	15.10	5.30	232.00	De	Se	Pl	In	D
Prosthemadera_novaeseelandiae	Passeriformes	9.50	12.10	1.72	105.83	De	Se	Pl	In	D
Protonotaria_citrea	Passeriformes	6.20	7.60	0.59	14.30	De	Mi	An	In	D
Prunella_modularis	Passeriformes	6.95	8.30	0.78	20.24	So	Se	Om	Te	D
Psaltriparus_minimus	Passeriformes	4.73	5.48	0.35	5.30	So	Se	An	In	D
Psarocolius_montezuma	Passeriformes	13.95	16.25	5.89	309.87	De	Se	An	In	D
Psephotus_haematonotus	Psittaciformes	7.70	9.53	1.97	61.39	So	Se	Pl	Te	D
Pseudeos_fuscata	Psittaciformes	8.80	12.35	4.20	149.00	So	Se	Pl	In	D
Psilopogon_pyrolophus	Piciformes	12.00	15.30	2.21	129.00	De	Se	Pl	In	D
Psittacella_brehmii	Psittaciformes	11.00	12.90	3.05	107.00	De	Se	Pl	In	D
Psittacula_krameri	Psittaciformes	9.80	11.80	3.90	116.11	So	Se	Pl	In	D
Psittacula_longicauda	Psittaciformes	10.30	12.45	3.75	107.00	De	Se	Pl	In	D
Psittacus_erithacus	Psittaciformes	13.20	15.90	9.18	333.00	De	Se	Pl	In	D

Psittrichas_fulgidus	Psittaciformes	13.90	17.50	7.80	864.99	De	Se	Pl	In	D
Psophia_crepitans	Gruiformes	17.20	21.88	5.85	1026.00	De	Se	Pl	Te	D
Psophia_leucoptera	Gruiformes	16.20	21.20	5.02	1315.58	De	Se	Pl	Te	D
Psophodes_cristatus	Passeriformes	7.85	9.35	1.40	40.71	So	Se	Om	Te	D
Psophodes_olivaceus	Passeriformes	10.00	11.60	2.18	62.36	De	Se	An	Ge	D
Pteridophora_alberti	Passeriformes	10.50	12.50	2.40	81.84	De	Pm	Pl	In	D
Pterocles_decoratus	Pterocliformes	10.30	13.25	1.31	181.34	Op	Pm	Pl	Te	D
Pterocles_lichtensteinii	Pterocliformes	10.50	13.45	1.13	209.10	Op	Se	Pl	Te	D
Pteroglossus_aracari	Piciformes	15.30	17.65	3.48	250.16	De	Se	Pl	In	D
Pteroglossus_bailloni	Piciformes	13.00	15.60	2.94	146.00	De	Se	Pl	In	D
Pteroglossus_torquatus	Piciformes	14.90	16.85	3.50	219.07	De	Se	Pl	In	D
Pteruthius_flaviscapis	Passeriformes	10.50	13.55	1.50	38.67	De	Se	An	In	D
Ptilinopus_jambu	Columbiformes	11.40	14.20	1.30	135.00	So	Se	Pl	In	D
Ptilinopus_rivoli	Columbiformes	10.80	13.50	1.45	149.00	So	Se	Pl	In	D
Ptilonorhynchus_violaceus	Passeriformes	14.70	17.28	4.72	218.91	De	Se	Om	In	D
Ptiloprora_guisei	Passeriformes	7.30	8.25	0.95	25.82	De	Se	An	In	D
Ptiloris_paradiseus	Passeriformes	10.50	12.25	2.78	119.49	De	Se	Om	In	D
Ptilorrhoa_caerulescens	Passeriformes	7.95	10.00	1.10	49.00	De	Se	An	Te	D
Puffinus_griseus	Procellariiformes	12.42	NA	5.31	787.00	Op	Mi	An	Ge	D
Pulsatrix_perspicillata	Strigiformes	32.80	NA	10.60	983.55	De	Se	An	In	N
Purpureicephalus_spurius	Psittaciformes	10.60	12.85	3.49	116.00	So	Se	Pl	In	D
Pycnonotus_barbatus	Passeriformes	8.30	9.85	1.16	32.10	So	Se	Pl	In	D
Pycnonotus_cafer	Passeriformes	8.60	10.05	1.18	42.85	So	Se	Pl	In	D
Pycnonotus_goiavier	Passeriformes	7.20	8.80	0.88	27.80	So	Se	Pl	In	D
Pycnonotus_jocosus	Passeriformes	8.10	9.00	0.93	29.47	So	Se	Pl	In	D
Pycnonotus_leucogenys	Passeriformes	7.90	9.45	0.79	35.94	So	Pm	Pl	In	D
Pycnoptilus_floccosus	Passeriformes	8.30	10.25	1.18	28.90	De	Se	An	Te	D
Pygochelidon_cyanoleuca	Passeriformes	6.70	7.88	0.43	9.70	Op	Pm	An	Ae	D
Pyriglena_leuconota	Passeriformes	8.40	10.30	0.93	32.30	De	Se	An	In	D
Pyrocephalus_rubinus	Passeriformes	7.40	8.98	0.46	14.40	Op	Mi	An	In	D
Pyroderus_scutatus	Passeriformes	17.20	21.80	4.30	357.00	De	Pm	Om	In	D
Pyrrhocorax_pyrrhocorax	Passeriformes	13.20	15.25	6.38	276.68	Op	Pm	An	Te	D

Pyrrholaemus_brunneus	Passeriformes	6.60	8.15	0.57	12.50	So	Se	Om	In	D
Pytilia_melba	Passeriformes	6.08	7.14	0.57	15.40	So	Se	Pl	Te	D
Pytilia_phoenicoptera	Passeriformes	5.90	6.95	0.36	14.50	So	Se	Pl	Te	D
Querula_purpurata	Passeriformes	13.40	15.40	2.13	107.35	De	Se	Om	In	D
Quiscalus_mexicanus	Passeriformes	10.60	12.60	2.96	160.47	Op	Se	Om	Te	D
Quiscalus_quiscula	Passeriformes	10.10	11.95	2.59	105.18	Op	Pm	An	Te	D
Ramphastos_dicolorus	Piciformes	15.80	19.00	4.53	331.00	De	Se	Pl	In	D
Ramphastos_sulfuratus	Piciformes	14.90	19.55	4.86	409.69	De	Se	Om	In	D
Ramphastos_toco	Piciformes	17.90	21.65	6.17	617.99	So	Se	Pl	In	D
Ramphastos_tucanus	Piciformes	16.10	19.25	5.98	659.58	De	Se	Pl	In	D
Ramphastos_vitellinus	Piciformes	15.90	18.45	4.81	360.36	De	Se	Om	In	D
Ramphocaenus_melanurus	Passeriformes	7.00	7.95	0.51	9.70	So	Se	An	In	D
Ramphocelus_carbo	Passeriformes	7.57	9.22	1.13	25.92	So	Se	Om	In	D
Ramsayornis_modestus	Passeriformes	5.40	6.50	0.51	12.10	So	Se	Om	In	D
Recurvirostra_americana	Charadriiformes	9.28	NA	2.10	304.48	Op	Pm	An	Te	D
Recurvirostra_avosetta	Charadriiformes	9.10	NA	1.83	304.00	Op	Pm	An	Te	D
Recurvirostra_novaehollandiae	Charadriiformes	10.70	NA	1.99	325.00	Op	Pm	An	Te	D
Regulus_calendula	Passeriformes	5.90	6.75	0.31	6.19	De	Mi	An	In	D
Regulus_ignicapilla	Passeriformes	5.00	6.25	0.35	5.60	De	Mi	An	In	D
Regulus_regulus	Passeriformes	4.60	5.95	0.35	5.54	De	Mi	An	In	D
Regulus_satrapa	Passeriformes	5.50	6.55	0.36	6.19	De	Mi	An	In	D
Reinwardtoena_reinwardtsi	Columbiformes	11.00	14.50	2.00	275.00	De	Se	Pl	In	D
Remiz_pendulinus	Passeriformes	4.80	6.15	0.43	9.30	So	Mi	Om	In	D
Rhagologus_leucostigma	Passeriformes	8.40	9.85	1.04	26.80	De	Se	Pl	In	D
Rhea_americana	Struthioniformes	27.94	33.59	18.88	23000.00	Op	Se	Om	Te	D
Rhea_pennata	Struthioniformes	14.40	18.23	18.53	23900.00	Op	Se	Pl	Te	D
Rhinocrypta_lanceolata	Passeriformes	9.50	11.50	1.40	61.90	So	Se	An	Te	D
Rhinopomastus_cyanomelas	Bucerotiformes	7.10	8.10	1.05	27.11	So	Se	An	In	D
Rhinopomastus_minor	Bucerotiformes	6.30	7.80	0.98	21.70	Op	Se	An	In	D
Rhinoptilus_chalcopterus	Charadriiformes	14.30	16.45	1.45	150.00	Op	Se	An	Te	D
Rhipidura_albicollis	Passeriformes	6.60	7.75	0.46	12.90	So	Se	An	In	D
Rhipidura_atra	Passeriformes	7.00	8.35	0.57	12.20	De	Se	An	In	D

Rhipidura_fuliginosa	Passeriformes	6.00	7.00	0.34	7.53	So	Se	An	In	D
Rhipidura_leucophrys	Passeriformes	7.80	9.90	0.58	27.44	Op	Se	An	In	D
Rhipidura_rufifrons	Passeriformes	6.20	7.40	0.36	10.08	So	Se	An	In	D
Rhipidura_rufiventris	Passeriformes	6.80	8.05	0.55	12.00	So	Se	An	In	D
Rhodinocichla_rosea	Passeriformes	8.90	10.90	1.35	48.00	De	Se	Pl	In	D
Rhodospingus_cruentus	Passeriformes	5.90	7.25	0.52	11.60	So	Se	Om	Ge	D
Rhyacornis_fuliginosa	Passeriformes	8.00	10.00	0.62	20.33	Op	Se	An	In	D
Rhynchopsitta_pachyrhyncha	Psittaciformes	11.30	13.85	11.20	324.77	De	Se	Pl	In	D
Rhynchotus_rufescens	Struthioniformes	13.40	19.40	3.66	843.47	Op	Se	Pl	Te	D
Rhynochetos_jubatus	Eurypygiformes	23.33	27.67	6.70	859.99	De	Se	An	Te	D
Riparia_riparia	Passeriformes	5.28	5.97	0.41	12.68	Op	Mi	An	Ae	D
Rollulus_rouloul	Galliformes	10.70	13.53	1.85	216.48	De	Se	Om	Te	D
Rostratula_benghalensis	Charadriiformes	10.20	NA	1.42	121.00	De	Pm	Om	Te	D
Rostrhamus_sociabilis	Accipitriformes	17.40	NA	5.09	366.94	So	Pm	An	In	D
Rupicola_peruvianus	Passeriformes	14.70	17.10	3.36	242.50	De	Se	Pl	In	D
Sagittarius_serpentarius	Accipitriformes	30.55	NA	15.00	4017.00	Op	Pm	An	Te	D
Salpinctes_obsoletus	Passeriformes	7.40	8.70	0.75	16.37	Op	Se	An	Te	D
Saltator_maximus	Passeriformes	9.10	10.95	1.39	47.62	So	Se	Pl	In	D
Sarcops_calvus	Passeriformes	10.80	14.05	2.80	142.00	De	Se	Om	In	D
Sarcoramphus_papa	Cathartiformes	27.00	NA	18.56	3400.00	Op	Se	Ca/Re	Te	D
Sasia_abnormis	Piciformes	5.80	7.15	0.58	9.20	De	Se	An	In	D
Saxicola_torquatus	Passeriformes	8.20	9.65	0.61	14.09	Op	Pm	An	In	D
Sayornis_nigricans	Passeriformes	7.70	8.90	0.49	18.63	So	Se	An	In	D
Sayornis_phoebe	Passeriformes	7.05	7.75	0.59	19.70	So	Mi	An	In	D
Sayornis_saya	Passeriformes	8.40	9.45	0.61	20.90	Op	Mi	An	In	D
Schiffornis_turdina	Passeriformes	9.95	12.15	1.07	31.70	De	Se	Om	In	D
Schistochlamys_melanopis	Passeriformes	8.70	9.85	1.22	33.00	So	Pm	Pl	In	D
Schoeniophylax_phryganophilus	Passeriformes	7.10	8.40	0.65	18.60	So	Se	An	In	D
Scissirostrum_dubium	Passeriformes	8.30	9.90	1.30	50.00	So	Pm	Pl	In	D
Scolopax_minor	Charadriiformes	11.70	NA	2.02	196.32	Op	Mi	An	Te	D
Scolopax_rusticola	Charadriiformes	11.53	NA	2.78	308.31	Op	Mi	An	Te	N
Scythrops_novaehollandiae	Cuculiformes	16.80	19.90	5.35	683.99	So	Mi	Om	In	D

Seiurus_aurocapilla	Passeriformes	7.30	9.15	0.70	18.80	De	Mi	An	Te	D
Seleucidis_melanoleucus	Passeriformes	12.40	14.15	3.41	174.43	De	Se	Pl	In	D
Semnornis_ramphastinus	Piciformes	10.30	13.90	1.91	97.70	De	Se	Om	In	D
Sericulus_chrysocephalus	Passeriformes	12.00	14.55	3.34	99.49	De	Se	Pl	In	D
Serinus_canaria	Passeriformes	6.30	7.15	0.68	24.30	So	Se	Pl	Ge	D
Serinus_mozambicus	Passeriformes	5.30	6.35	0.48	11.87	Op	Se	Pl	Ge	D
Serinus_serinus	Passeriformes	4.60	5.90	0.46	11.20	So	Pm	Pl	Ge	D
Setophaga_ruticilla	Passeriformes	6.18	7.29	0.36	8.24	De	Mi	An	In	D
Sicalis_flaveola	Passeriformes	6.70	7.40	0.70	16.89	So	Se	Pl	Te	D
Sitta_canadensis	Passeriformes	6.30	7.30	0.57	9.80	De	Mi	Om	In	D
Sitta_europaea	Passeriformes	7.00	8.75	1.02	20.37	De	Se	An	In	D
Sitta_pygmaea	Passeriformes	5.60	6.55	0.55	10.60	De	Se	Om	In	D
Sittasomus_griseicapillus	Passeriformes	6.40	8.05	0.55	13.12	De	Se	An	In	D
Smicrornis_brevirostris	Passeriformes	4.80	6.20	0.29	5.30	So	Se	An	In	D
Smithornis_capensis	Passeriformes	10.00	12.00	0.78	23.20	So	Se	An	In	D
Somateria_mollissima	Anseriformes	13.40	NA	8.32	2060.93	Op	Pm	An	Aq	D
Spermophaga_haematina	Passeriformes	7.35	8.95	0.88	23.00	De	Se	Pl	In	D
Sphecotheres_vieilloti	Passeriformes	11.30	13.80	2.24	121.50	So	Se	Pl	In	D
Spheniscus_humboldti	Sphenisciformes	20.50	NA	15.98	4366.39	Op	Pm	An	Aq	D
Spheniscus_magellanicus	Sphenisciformes	27.30	NA	13.49	4105.10	Op	Pm	An	Aq	D
Sphyrapicus_nuchalis	Piciformes	7.70	9.90	1.27	48.49	So	Mi	Om	In	D
Sphyrapicus_varius	Piciformes	8.37	10.18	1.30	50.30	De	Mi	Om	In	D
Spindalis_zena	Passeriformes	7.00	8.75	1.31	25.36	So	Se	Pl	In	D
Spiza_americana	Passeriformes	7.00	8.50	0.91	26.18	Op	Mi	Om	Te	D
Spizaetus_ornatus	Accipitriformes	24.10	NA	7.60	1197.40	De	Se	An	Ge	D
Spizella_arborea	Passeriformes	6.00	7.00	0.74	17.83	So	Mi	Om	Te	D
Stachyris_chrysaea	Passeriformes	5.20	6.25	0.43	9.00	De	Se	An	In	D
Stachyris_nigriceps	Passeriformes	6.40	7.65	0.76	15.80	De	Se	An	In	D
Steatornis_caripensis	Caprimulgiformes	15.47	NA	3.90	408.00	De	Se	Pl	In	N
Stelgidopteryx_ruficollis	Passeriformes	7.20	8.20	0.53	16.10	Op	Se	An	Ae	D
Stephanoaetus_coronatus	Accipitriformes	28.70	NA	14.06	3640.00	De	Se	An	Ge	D
Sterna_hirundo	Charadriiformes	13.70	NA	1.88	129.15	Op	Mi	An	Ae	D

Stigmatopelia_chinensis	Columbiformes	8.30	11.30	1.35	159.00	Op	Se	Pl	Te	D
Stigmatopelia_senegalensis	Columbiformes	8.30	11.50	1.24	82.13	Op	Se	Pl	Te	D
Stigmatura_budytoides	Passeriformes	6.80	7.60	0.53	11.08	So	Se	An	In	D
Stiltia_isabella	Charadriiformes	10.20	13.05	1.05	65.50	Op	Pm	An	Te	D
Stipiturus_malachurus	Passeriformes	5.30	6.35	0.40	7.30	Op	Se	An	Ge	D
Strepera_graculina	Passeriformes	15.30	19.45	5.34	298.86	So	Se	Om	Ge	D
Strepera_versicolor	Passeriformes	16.50	20.00	5.97	395.47	So	Se	An	Te	D
Streptopelia_decaocto	Columbiformes	9.30	12.25	1.52	148.96	Op	Se	Pl	Te	D
Streptopelia_turtur	Columbiformes	9.30	12.20	1.39	132.00	So	Mi	Pl	Te	D
Streptoprocne_zonaris	Caprimulgiformes	12.95	15.25	1.48	86.79	De	Pm	An	Ae	D
Strigops_habroptila	Psittaciformes	13.45	15.40	16.50	1732.05	So	Se	Pl	Ge	N
Strix_aluco	Strigiformes	24.96	NA	9.08	472.46	De	Se	An	In	N
Strix_nebulosa	Strigiformes	14.00	NA	14.66	1061.89	So	Se	An	In	N
Strix_uralensis	Strigiformes	25.60	NA	11.21	780.56	So	Se	An	In	N
Strix_varia	Strigiformes	28.00	NA	12.55	711.49	So	Se	An	In	N
Struthidea_cinerea	Passeriformes	11.00	12.48	2.98	132.00	So	Se	Om	Te	D
Struthio_camelus	Struthioniformes	38.93	47.79	40.23	111000.00	Op	Se	Pl	Te	D
Sturnella_magna	Passeriformes	8.75	9.88	2.22	91.76	Op	Pm	An	Te	D
Sturnella_militaris	Passeriformes	8.00	9.63	1.12	48.01	Op	Pm	An	Te	D
Sturnella_neglecta	Passeriformes	10.70	12.50	1.94	100.06	Op	Pm	Om	Te	D
Sturnus_contra	Passeriformes	8.90	10.55	1.77	83.99	Op	Se	Om	Ge	D
Sturnus_vulgaris	Passeriformes	6.97	7.79	1.97	77.14	Op	Pm	Om	In	D
Surnia_ulula	Strigiformes	20.50	NA	7.48	319.90	Op	Pm	An	In	D
Sylvia_atricapilla	Passeriformes	6.40	8.40	0.65	16.70	De	Mi	Om	In	D
Sylvia_borin	Passeriformes	7.00	8.45	0.62	18.20	De	Mi	Om	In	D
Syma_megarhyncha	Coraciiformes	11.90	14.00	1.35	56.00	De	Se	An	In	D
Syma_torotoro	Coraciiformes	10.40	12.60	1.11	37.70	De	Se	An	In	D
Synallaxis_brachyura	Passeriformes	7.00	8.35	0.86	18.30	So	Se	An	In	D
Syrmaticus_reevesii	Galliformes	15.80	19.80	4.38	1204.58	So	Se	Pl	Te	D
Syrrhaptes_paradoxus	Pterocliformes	8.80	12.10	1.54	256.43	Op	Pm	Pl	Te	D
Tachornis_phoenicobia	Caprimulgiformes	7.60	9.00	0.28	10.20	De	Se	An	Ae	D
Tachycineta_bicolor	Passeriformes	8.20	9.05	0.55	21.20	Op	Mi	An	Ae	D

Tachymarptis_melba	Caprimulgiformes	12.10	14.15	1.09	100.38	De	Mi	An	Ae	D
Taeniopygia_bichenovii	Passeriformes	4.70	5.80	0.40	9.60	So	Se	Pl	Te	D
Taeniopygia_guttata	Passeriformes	5.20	6.03	0.44	12.04	So	Pm	Pl	Te	D
Tangara_cayana	Passeriformes	7.00	8.60	0.80	18.00	So	Se	Pl	In	D
Tanygnathus_lucionensis	Psittaciformes	13.00	15.10	5.71	215.00	De	Se	Pl	In	D
Tanysiptera_galatea	Coraciiformes	11.80	14.75	1.43	50.00	De	Se	An	In	D
Tanysiptera_sylvia	Coraciiformes	13.00	14.95	1.14	49.60	De	Mi	An	In	D
Tapera_naevia	Cuculiformes	9.00	12.75	0.89	48.42	Op	Se	An	Ge	D
Taraba_major	Passeriformes	9.40	11.68	1.52	59.20	So	Se	An	In	D
Tauraco_corythaix	Musophagiformes	13.45	16.98	2.70	308.00	De	Se	Pl	In	D
Tauraco_erythrolophus	Musophagiformes	13.30	15.90	2.34	261.24	So	Se	Pl	In	D
Tauraco_hartlaubi	Musophagiformes	13.05	16.23	3.35	224.00	De	Se	Pl	In	D
Tauraco_leucotis	Musophagiformes	13.15	15.90	3.35	265.00	De	Se	Pl	In	D
Tchagra_australis	Passeriformes	9.30	10.80	1.23	32.30	So	Se	An	Te	D
Telophorus_zeylonus	Passeriformes	10.60	12.30	1.91	65.02	So	Se	An	Te	D
Tephrodornis_pondicerianus	Passeriformes	8.30	9.90	0.89	20.20	So	Se	An	In	D
Terathopius_ecaudatus	Accipitriformes	33.00	NA	14.69	2200.00	So	Se	An	Ge	D
Tersina_viridis	Passeriformes	7.63	9.35	0.93	29.00	So	Se	Pl	In	D
Tetrao_tetrix	Galliformes	10.60	13.05	3.80	1068.66	Op	Se	Pl	Te	D
Tetrao_urogallus	Galliformes	20.40	24.50	6.14	2716.61	So	Se	Pl	Ge	D
Thalurania_furcata	Caprimulgiformes	4.60	5.50	0.20	4.19	De	Se	Pl	Ae	D
Thamnomanes_caesius	Passeriformes	8.00	9.30	0.61	15.70	De	Se	An	In	D
Thamnophilus_caerulescens	Passeriformes	7.80	9.75	0.98	21.10	De	Se	An	In	D
Thamnophilus_punctatus	Passeriformes	8.60	10.55	0.94	19.40	De	Se	An	In	D
Thinocorus_orbignyianus	Charadriiformes	8.80	NA	1.16	115.00	Op	Se	Pl	Te	D
Thinocorus_rumicivorus	Charadriiformes	7.35	NA	0.72	53.10	Op	Se	Pl	Te	D
Thraupis_episcopus	Passeriformes	8.00	9.60	1.11	35.00	So	Se	Om	In	D
Thryothorus_nigricapillus	Passeriformes	7.80	9.35	1.06	23.90	De	Se	An	In	D
Tiaris_olivaceus	Passeriformes	5.40	6.45	0.48	8.50	So	Pm	Pl	In	D
Tinamus_major	Struthioniformes	13.90	18.98	2.89	1026.21	De	Se	Pl	Te	D
Tityra_cayana	Passeriformes	12.30	14.15	1.61	68.10	So	Se	Pl	In	D
Tityra_semifasciata	Passeriformes	12.40	15.05	1.88	79.30	De	Se	Pl	In	D

Tockus_deckeni	Bucerotiformes	12.40	14.85	3.45	167.72	Op	Se	An	Te	D
Tockus_erythrorhynchus	Bucerotiformes	11.90	14.60	3.53	138.56	Op	Se	An	Te	D
Tockus_fasciatus	Bucerotiformes	15.30	18.50	4.80	258.83	De	Se	Om	In	D
Todiramphus_chloris	Coraciiformes	10.50	13.95	1.34	66.09	So	Se	An	In	D
Todiramphus_macleayii	Coraciiformes	10.40	12.75	1.07	37.30	So	Mi	An	In	D
Todiramphus_pyrrhopygius	Coraciiformes	10.60	13.13	1.13	51.70	So	Se	An	In	D
Todiramphus_sanctus	Coraciiformes	9.30	12.05	1.03	52.96	So	Mi	An	In	D
Todirostrum_cinereum	Passeriformes	5.97	6.72	0.31	6.29	So	Se	An	In	D
Todus_mexicanus	Coraciiformes	6.80	7.90	0.40	5.90	De	Se	An	In	D
Todus_subulatus	Coraciiformes	6.80	7.55	0.43	8.70	So	Se	An	In	D
Todus_todus	Coraciiformes	6.87	8.20	0.41	6.40	De	Se	An	In	D
Tolmomyias_sulphurescens	Passeriformes	8.30	9.30	0.51	14.30	De	Se	An	In	D
Toxorhamphus_iliolophus	Passeriformes	5.50	6.75	0.48	11.00	De	Se	Om	In	D
Toxorhamphus_poliopterus	Passeriformes	5.60	6.80	0.43	11.35	So	Se	Om	In	D
Toxostoma_curvirostre	Passeriformes	11.00	12.85	2.13	80.54	Op	Se	Om	Te	D
Toxostoma_rufum	Passeriformes	9.38	10.99	2.00	68.80	De	Pm	Om	Ge	D
Tregellasia_capito	Passeriformes	8.50	10.60	0.77	13.90	De	Se	An	In	D
Tregellasia_leucops	Passeriformes	8.50	10.60	0.59	15.57	De	Se	An	In	D
Treron_apicauda	Columbiformes	9.45	12.15	1.40	217.19	So	Se	Pl	In	D
Treron_calvus	Columbiformes	10.20	13.10	1.58	197.53	So	Pm	Pl	In	D
Treron_pompadora	Columbiformes	10.20	12.70	1.39	234.00	So	Se	Pl	In	D
Treron_vernans	Columbiformes	8.33	NA	1.25	132.00	So	Se	Pl	In	D
Trichoglossus_chlorolepidotus	Psittaciformes	8.70	10.40	3.05	87.10	So	Se	Pl	In	D
Trichoglossus_haematodus	Psittaciformes	8.90	10.70	3.66	113.25	De	Se	Pl	In	D
Tricholaema_leucomelas	Piciformes	8.30	10.05	0.98	32.20	So	Se	Pl	In	D
Trichothraupis_melanops	Passeriformes	8.40	9.90	0.90	22.58	De	Se	An	In	D
Triclaria_malachitacea	Psittaciformes	11.60	13.45	4.28	90.00	De	Se	Pl	In	D
Tringa_glareola	Charadriiformes	8.80	NA	1.04	62.05	Op	Mi	An	Te	D
Tringa_melanoleuca	Charadriiformes	11.80	NA	1.79	161.74	Op	Mi	An	Te	D
Tringa_solitaria	Charadriiformes	8.20	NA	0.82	48.40	Op	Mi	An	Te	D
Tringa_totanus	Charadriiformes	10.00	NA	1.52	129.00	Op	Mi	An	Te	D
Trochilus_polytmus	Caprimulgiformes	4.20	5.10	0.19	4.84	De	Se	Pl	Ae	D

Troglodytes_aedon	Passeriformes	5.00	5.84	0.53	10.85	Op	Se	An	In	D
Troglodytes_troglodytes	Passeriformes	6.15	7.80	0.48	9.74	De	Mi	An	In	D
Trogon_citreolus	Trogoniformes	12.30	14.00	1.48	79.00	So	Se	Om	In	D
Trogon_curucui	Trogoniformes	12.40	14.72	0.89	54.00	De	Se	An	In	D
Trogon_massena	Trogoniformes	14.40	17.05	2.13	141.00	De	Se	Pl	In	D
Trogon_melanurus	Trogoniformes	13.00	15.90	1.86	114.00	De	Se	Pl	In	D
Trogon_violaceus	Trogoniformes	12.40	14.35	1.41	46.50	De	Se	Pl	In	D
Trogon_viridis	Trogoniformes	12.15	14.63	1.79	89.69	De	Se	Om	In	D
Tryngites_subruficollis	Charadriiformes	9.10	NA	1.03	62.10	Op	Mi	An	Te	D
Turdus_falcklandii	Passeriformes	12.10	14.25	2.34	93.89	So	Pm	Om	Ge	D
Turdus_fuscater	Passeriformes	11.20	14.85	2.69	143.00	So	Se	Om	Ge	D
Turdus_merula	Passeriformes	10.70	12.85	1.90	102.73	So	Se	Om	Ge	D
Turdus_migratorius	Passeriformes	9.80	13.00	1.64	78.50	Op	Mi	Om	Ge	D
Turdus_philomelos	Passeriformes	11.00	NA	1.63	67.74	De	Mi	Om	Ge	D
Turdus_rufiventris	Passeriformes	10.60	12.90	1.33	69.44	De	Pm	Om	Ge	D
Turdus_viscivorus	Passeriformes	12.30	14.35	2.31	117.37	So	Se	Om	In	D
Turnix_melanogaster	Charadriiformes	8.70	10.90	1.06	87.06	De	Se	An	Te	D
Turnix_nigricollis	Charadriiformes	7.00	9.95	0.87	61.30	So	Se	Om	Te	D
Turnix_suscitator	Charadriiformes	7.00	10.15	0.71	49.10	Op	Se	Pl	Te	D
Turnix_sylvaticus	Charadriiformes	5.15	7.54	0.58	52.69	So	Se	Om	Te	D
Turnix_varius	Charadriiformes	7.60	10.85	1.04	90.23	So	Se	Pl	Te	D
Turnix_velox	Charadriiformes	6.00	9.40	0.67	44.28	Op	Se	Pl	Te	D
Turtur_afer	Columbiformes	8.15	11.23	0.87	65.60	So	Se	Pl	Te	D
Turtur_tympanistria	Columbiformes	8.40	11.45	0.97	70.58	So	Se	Pl	Te	D
Tyrannus_dominicensis	Passeriformes	9.10	11.40	1.00	46.50	Op	Pm	An	In	D
Tyrannus_forficatus	Passeriformes	8.80	10.75	0.86	39.30	So	Mi	Om	In	D
Tyrannus_melancholicus	Passeriformes	8.37	9.73	0.90	37.40	Op	Se	An	In	D
Tyrannus_tyrannus	Passeriformes	8.40	9.70	0.95	39.85	So	Mi	An	In	D
Tyrannus_verticalis	Passeriformes	9.35	10.80	0.98	39.60	Op	Mi	An	In	D
Tyto_alba	Strigiformes	17.80	NA	6.51	403.32	Op	Se	An	In	N
Tyto_capensis	Strigiformes	15.60	NA	5.23	364.74	Op	Pm	An	Ge	N
Tyto_tenebricosa	Strigiformes	28.80	NA	12.70	660.60	De	Se	An	Ge	N

Upupa_epops	Bucerotiformes	8.48	10.25	1.35	66.93	Op	Mi	An	Te	D
Uraeginthus_bengalus	Passeriformes	5.40	6.50	0.43	9.90	Op	Se	Pl	Te	D
Urocissa_erythrorhyncha	Passeriformes	14.60	17.45	3.67	151.52	De	Se	An	In	D
Urocolius_indicus	Coliiformes	7.87	9.42	1.07	52.70	So	Se	Pl	In	D
Urocolius_macrourus	Coliiformes	7.20	9.15	1.00	45.50	So	Se	Pl	In	D
Vanellus_chilensis	Charadriiformes	14.70	NA	2.46	327.00	Op	Se	An	Te	D
Vanellus_miles	Charadriiformes	14.00	NA	2.85	387.00	Op	Se	An	Te	D
Vanellus_senegallus	Charadriiformes	14.50	NA	2.43	248.00	Op	Se	An	Te	D
Vanellus_vanellus	Charadriiformes	12.60	NA	2.33	218.37	Op	Pm	An	Te	D
Veniliornis_passerinus	Piciformes	8.10	9.75	1.45	32.10	So	Se	An	In	D
Vermivora_peregrina	Passeriformes	4.90	6.35	0.43	8.90	De	Mi	An	In	D
Vermivora_ruficapilla	Passeriformes	4.50	6.30	0.40	8.09	De	Mi	An	In	D
Vidua_macroura	Passeriformes	5.90	7.23	0.53	15.74	Op	Se	Pl	Te	D
Vidua_paradisaea	Passeriformes	6.00	7.80	0.64	20.35	Op	Se	Pl	Te	D
Vireo_altiloquus	Passeriformes	7.10	8.55	0.75	18.97	So	Se	Om	In	D
Vireo_griseus	Passeriformes	6.30	8.65	0.58	11.40	So	Mi	An	In	D
Vireo_magister	Passeriformes	7.80	8.75	0.72	15.40	De	Se	Om	In	D
Vireo_olivaceus	Passeriformes	6.80	8.30	0.61	16.06	De	Mi	Om	In	D
Vireo_philadelphicus	Passeriformes	6.80	8.08	0.46	11.50	De	Mi	An	In	D
Vireo_solitarius	Passeriformes	7.60	9.75	0.63	15.30	De	Mi	An	In	D
Volatinia_jacarina	Passeriformes	5.60	6.65	0.49	9.94	Op	Se	Pl	Te	D
Vultur_gryphus	Cathartiformes	23.70	NA	31.56	11236.10	Op	Pm	Ca/Re	Te	D
Willisornis_poecilinotus	Passeriformes	8.55	9.88	0.64	18.40	De	Se	An	In	D
Xanthocephalus_xanthocephalus	Passeriformes	7.67	8.53	1.65	62.68	So	Pm	Pl	Te	D
Xanthomyza_phrygia	Passeriformes	7.60	9.75	1.03	39.99	So	Pm	Pl	In	D
Xenops_minutus	Passeriformes	5.28	6.59	0.49	10.60	De	Se	An	In	D
Xenus_cinereus	Charadriiformes	8.40	NA	1.03	78.80	Op	Mi	An	Te	D
Xiphorhynchus_guttatus	Passeriformes	8.50	10.15	1.50	59.69	De	Se	An	In	D
Xolmis_irupero	Passeriformes	9.00	11.10	0.82	28.70	Op	Pm	An	In	D
Xolmis_pyrope	Passeriformes	10.50	12.55	1.28	35.30	So	Pm	An	In	D
Zenaida_macroura	Columbiformes	8.06	10.49	1.19	118.93	Op	Se	Pl	Te	D
Zonotrichia_albicollis	Passeriformes	6.80	8.85	1.05	24.40	So	Mi	Pl	Te	D

Zonotrichia_capensis	Passeriformes	6.80	8.30	0.87	20.31	Op	Se	Pl	Ge	D
Zonotrichia_leucophrys	Passeriformes	5.50	7.23	0.97	28.00	So	Mi	Om	Ge	D
Zosterops_japonicus	Passeriformes	5.10	6.55	0.54	11.29	So	Se	Om	In	D
Zosterops_lateralis	Passeriformes	5.00	6.65	0.47	12.68	So	Se	Om	In	D

Table S2 Details of species traits and the number of species sampled in our dataset. References are listed at the end of this document.

Variable	Sample size	Sources
Axial length (mm)	1274	[14−16, 25, 95]
Transverse diameter (mm)	1041	[25]
Brain volume (ml)	1274	[96−103]
Body mass (g)	1274	[55]
Habitat openness	1274	[55]
Migration	1274	[55]
Food type	1274	[55]
Foraging habitat	1274	[55]
Activity pattern	1274	[15, 40, 104−111]

Table S3 Five separate MCMCglmm models to test for interactions between relative eye volume with either ecological and behavioral variables (e.g., eye volume~habitat openness + body mass). Significant predictors are marked in bold.

Variables	Posterior mean	Lower 95% CI	Upper 95% CI	P_{mcmc}
(a)				
(Intercept)	−1.400	−1.563	−1.240	<0.001
Habitat openness (dense habitat vs. open habitat)	−0.063	−0.089	−0.037	**<0.001**
Habitat openness (dense habitat vs. semiopen habitat)	−0.031	−0.048	−0.012	**<0.001**
Body mass	0.725	0.697	0.750	<0.001
(b)				
(Intercept)	−1.438	−1.604	−1.262	<0.001
Migration (migratory vs. partially migratory)	0.008	−0.026	0.038	0.615
Migration (migratory vs. sedentary)	0.024	−0.001	0.050	0.061
Body mass	0.721	0.695	0.748	<0.001
(c)				
(Intercept)	−1.385	−1.554	−1.226	<0.001
Food type (animals vs. plants)	−0.081	−0.114	−0.049	**<0.001**
Food type (animals vs. omnivorous)	−0.039	−0.067	−0.011	**0.006**
Body mass	0.731	0.705	0.756	<0.001
(d)				
(Intercept)	−1.422	−1.607	−1.232	<0.001
Foraging habitat (aerial vs. generalist)	−0.022	−0.117	0.064	0.619
Foraging habitat (aerial vs. insessorial)	0.022	−0.068	0.108	0.631
Foraging habitat (aerial vs. terrestrial)	−0.032	−0.124	0.055	0.479
Body mass	0.731	0.705	0.757	<0.001
(e)				
(Intercept)	−1.413	−1.580	−1.245	<0.001
Activity pattern (diurnal vs. nocturnal)	−0.102	−0.237	0.023	0.126
Body mass	0.723	0.697	0.750	<0.001

Table S4 Five separate MCMCglmm models to test for interactions between relative axial length with either ecological and behavioral variables (e.g., axial length~habitat openness + body mass). Significant predictors are marked in bold.

Variables	Posterior mean	Lower 95% CI	Upper 95% CI	P_{mcmc}
(a)				
(Intercept)	0.487	0.422	0.556	<0.001
Habitat openness (dense habitat vs. open habitat)	−0.023	−0.032	−0.013	**<0.001**
Habitat openness (dense habitat vs. semiopen habitat)	−0.010	−0.017	−0.002	**0.004**
Body mass	0.235	0.226	0.244	<0.001
(b)				
(Intercept)	0.476	0.411	0.540	<0.001
Migration (migratory vs. partially migratory)	−0.005	−0.015	0.006	0.325
Migration (migratory vs. sedentary)	0.005	−0.005	0.013	0.292
Body mass	0.234	0.225	0.243	<0.001
(c)				
(Intercept)	0.488	0.422	0.554	<0.001
Food type (animals vs. plants)	−0.029	−0.042	−0.017	**<0.001**
Food type (animals vs. omnivorous)	−0.015	−0.025	−0.004	**0.007**
Food type (animals vs. carrion or refuse)	−0.072	−0.127	−0.010	**0.013**
Body mass	0.237	0.228	0.246	<0.001
(d)				
(Intercept)	0.489	0.423	0.560	<0.001
Foraging habitat (aerial vs. aquatic)	−0.060	−0.110	−0.009	**0.024**
Foraging habitat (aerial vs. generalist)	−0.017	−0.036	0.001	0.072
Foraging habitat (aerial vs. insessorial)	−0.002	−0.022	0.016	0.807
Foraging habitat (aerial vs. terrestrial)	−0.022	−0.040	−0.001	**0.032**
Body mass	0.237	0.228	0.247	<0.001
(e)				
(Intercept)	0.476	0.410	0.544	<0.001
Activity pattern (diurnal vs. nocturnal)	0.035	0.006	0.067	**0.022**
Body mass	0.233	0.224	0.242	<0.001

Table S5 Phylogenetically controlled mixed models in MCMCglmm assessing the effect of ecological factors and behaviours on the ratio (axial length/transverse diameter) in 1041 species of birds.

Variables	Posterior mean	Lower 95% CI	Upper 95% CI	P_{mcmc}
(a)				
(Intercept)	−0.094	−0.123	−0.066	<0.001
Habitat openness (dense habitat vs. open habitat)	0.0005	−0.005	0.006	0.849
Habitat openness (dense habitat vs. semiopen habitat)	−0.0001	−0.004	0.004	0.940
(b)				
(Intercept)	−0.092	−0.121	−0.063	<0.001
Migration (migratory vs. partially migratory)	−0.003	−0.010	0.003	0.315
Migration (migratory vs. sedentary)	−0.002	−0.007	0.004	0.532
(c)				
(Intercept)	−0.091	−0.120	−0.064	<0.001
Food type (animals vs. plants)	−0.004	−0.010	0.002	0.197
Food type (animals vs. omnivorous)	−0.0003	−0.006	0.005	0.887
(d)				
(Intercept)	−0.094	−0.126	−0.059	<0.001
Foraging habitat (aerial vs. generalist)	0.002	−0.017	0.019	0.810
Foraging habitat (aerial vs. insessorial)	0.0005	−0.018	0.017	0.960
Foraging habitat (aerial vs. terrestrial)	0.0006	−0.018	0.018	0.949
(e)				
(Intercept)	−0.095	−0.124	−0.066	<0.001
Activity pattern (diurnal vs. nocturnal)	0.020	−0.005	0.045	0.107

Table S6 Phylogenetically controlled mixed models in MCMCglmm assessing the effect of relative brain size and ecological variables on relative eye volume in 1041 species of birds. Significant predictors are marked in bold.

Variables	Posterior mean	Lower 95% CI	Upper 95% CI	P_{mcmc}
(Intercept)	−1.343	−1.512	−1.181	<0.001
Relative brain size	0.346	0.229	0.455	**<0.001**
Habitat openness (dense habitat vs. open habitat)	−0.057	−0.083	−0.032	**<0.001**
Habitat openness (dense habitat vs. semiopen habitat)	−0.025	−0.043	−0.007	**0.009**
Food type (animals vs. plants)	−0.079	−0.111	−0.047	**<0.001**
Food type (animals vs. omnivorous)	−0.038	−0.066	−0.010	**0.009**
Body mass	0.724	0.696	0.749	<0.001

Table S7 Phylogenetically controlled mixed models in MCMCglmm assessing the effect of relative brain size, ecological and behavioral variables on relative axial length in 1274 species of birds. Significant predictors are marked in bold.

Variables	Posterior mean	Lower 95% CI	Upper 95% CI	P_{mcmc}
(Intercept)	0.496	0.433	0.557	<0.001
Relative brain size	0.156	0.116	0.195	**<0.001**
Habitat openness (dense habitat vs. open habitat)	−0.019	−0.028	−0.010	**<0.001**
Habitat openness (dense habitat vs. semiopen habitat)	−0.008	−0.014	−0.001	**0.019**
Food type (animals vs. plants)	−0.028	−0.040	−0.016	**<0.001**
Food type (animals vs. omnivorous)	−0.014	−0.024	−0.004	**0.008**
Food type (animals vs. carrion or refuse)	−0.062	−0.116	−0.003	**0.029**
Activity pattern (diurnal vs. nocturnal)	0.022	−0.007	0.052	0.155
Body mass	0.236	0.227	0.245	<0.001

Table S8 Diagnostic values for assessing our two multipredictor models for MCMCglmm analyses. Values for (a) eye volume~relative brain size + habitat openness + food type + body mass, and (b) axial length~relative brain size + habitat openness + food type + activity pattern + body mass.

Factors	Upper confidence interval for Gelman and Rubin's Convergence Diagnostic	Effective sample size	Geweke's Convergence Diagnostic
**(a)**			
(Intercept)	1	4000	0.554
Relative brain size	1	3425	0.008
Habitat openness (dense habitat vs. open habitat)	1	4000	−0.759
Habitat openness (dense habitat vs. semiopen habitat)	1	4000	1.780
Food type (animals vs. plants)	1	4000	1.188
Food type (animals vs. omnivorous)	1	4000	0.760
Body mass	1	4479	−1.651
**(b)**			
(Intercept)	1	4221	−0.742
Relative brain size	1	3615	−0.499
Habitat openness (dense habitat vs. open habitat)	1	4000	0.074
Habitat openness (dense habitat vs. semiopen habitat)	1	3716	−0.511
Food type (animals vs. plants)	1	4224	−1.120
Food type (animals vs. omnivorous)	1	4000	−0.293
Food type (animals vs. carrion or refuse)	1	4000	0.779
Activity pattern (diurnal vs. nocturnal)	1	4000	0.512
Body mass	1	3810	0.534

Figures S1 to S4

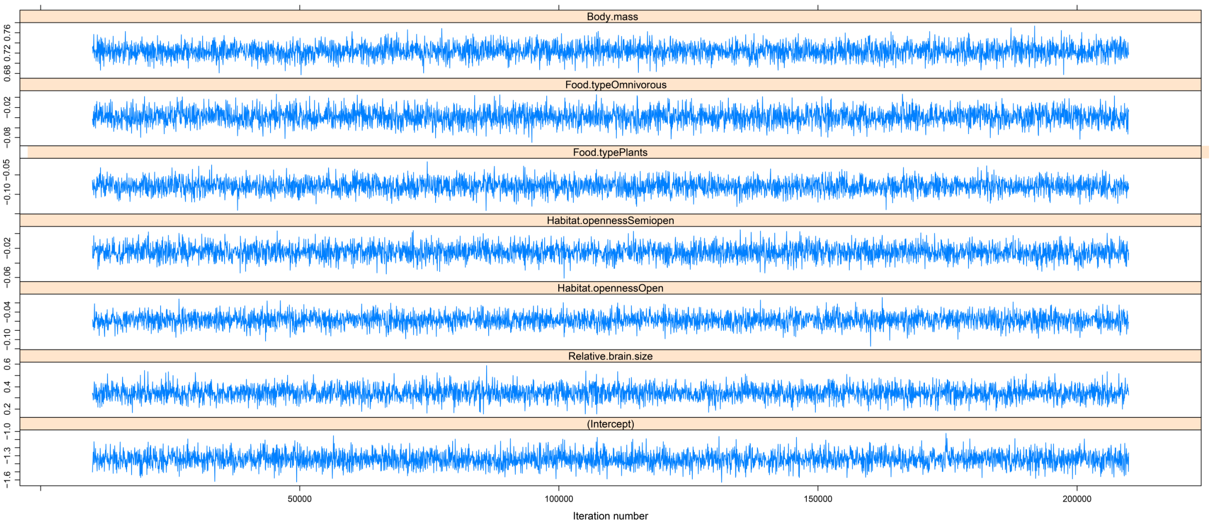

Fig. S1 Trace plots for fixed factors in the first multipredictor model (eye volume~relative brain size + habitat openness + food type + body mass). See Table S8 for additional diagnostics.

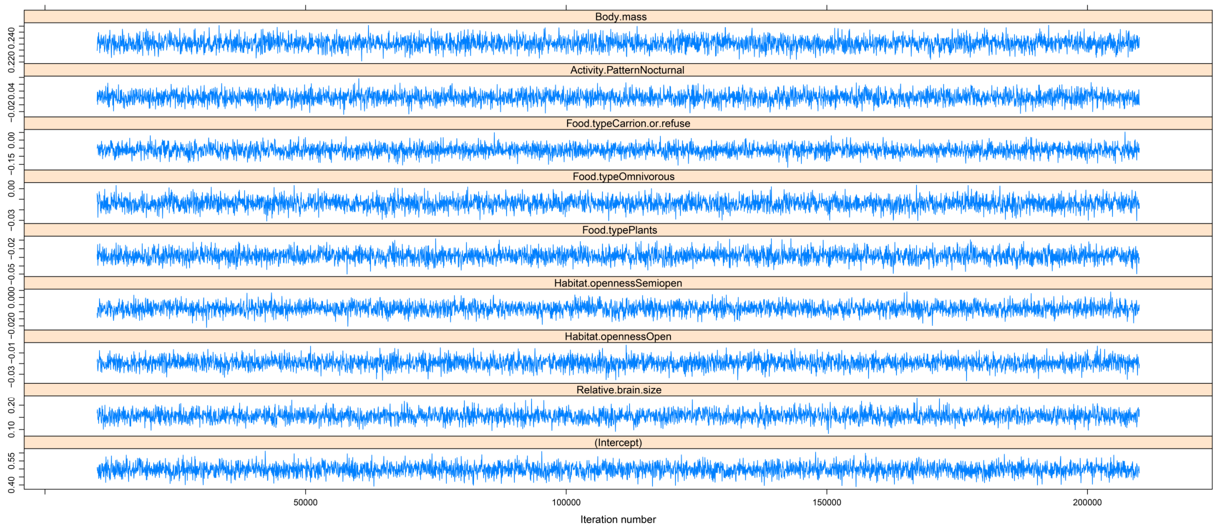

Fig. S2 Trace plots for fixed factors in the second multipredictor model (axial length~relative brain size + habitat openness + food type + activity pattern + body mass). See Table S8 for additional diagnostics.

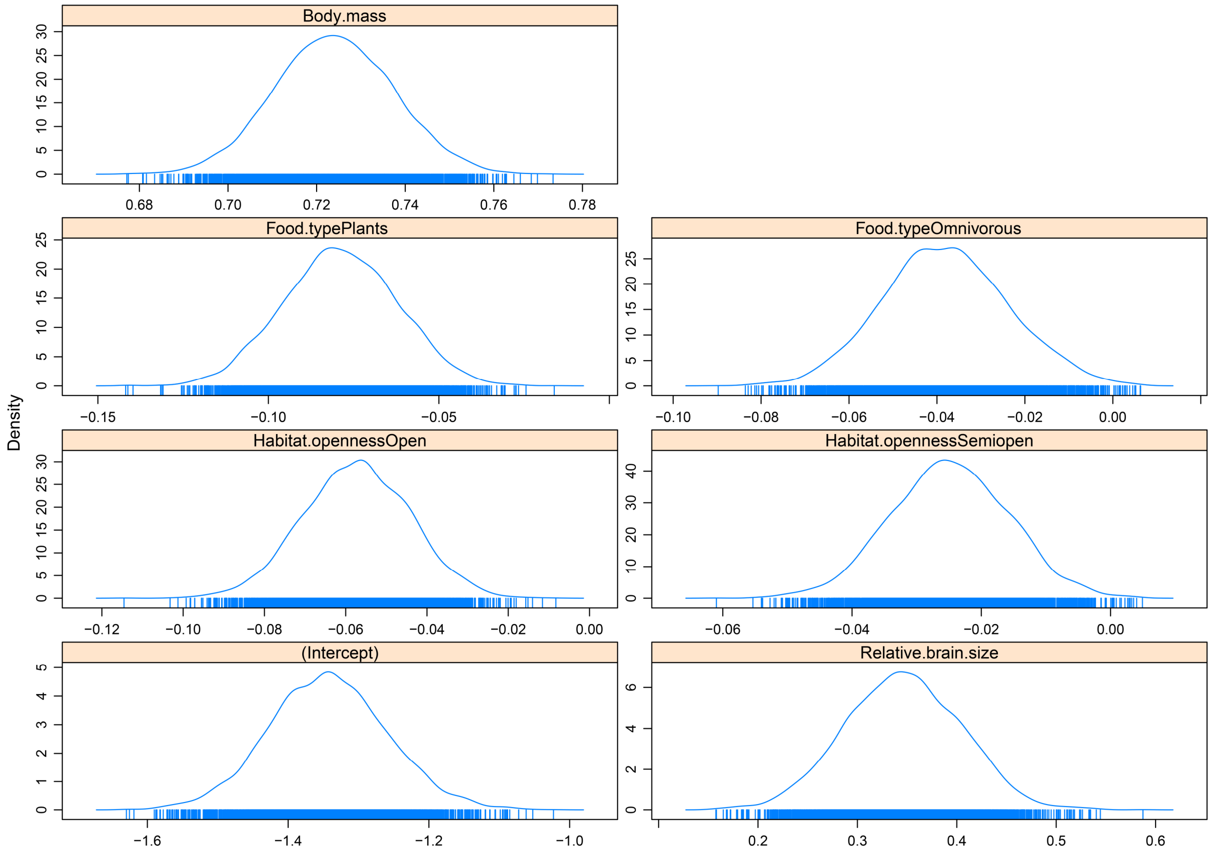

Fig. S3 Density plots for fixed factors in the first multipredictor model (eye volume~relative brain size + habitat openness + food type + body mass). See Table S8 for additional diagnostics.

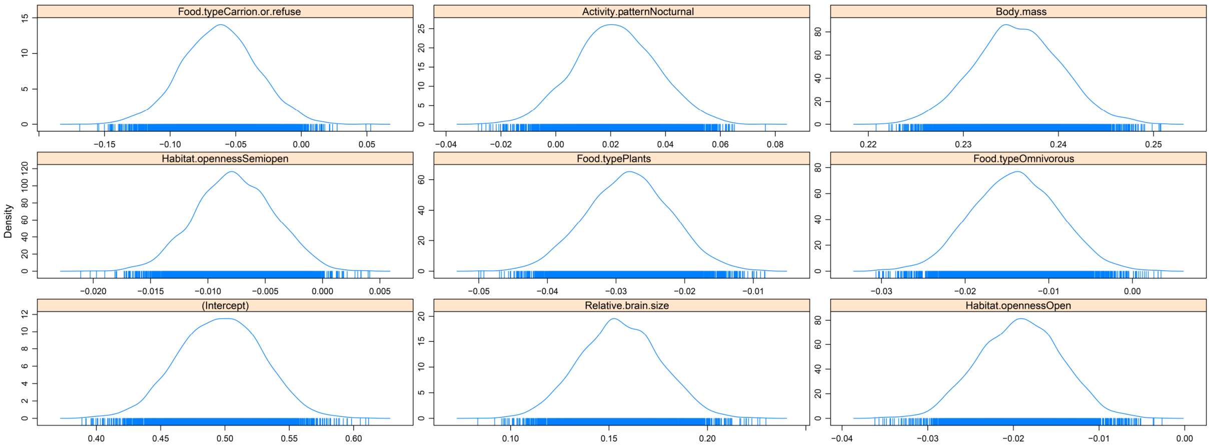

Fig. S4 Density plots for fixed factors in the second multipredictor model (axial length~relative brain size + habitat openness + food type + activity pattern + body mass). See Table S8 for additional diagnostics.
